# A colon mimetic screening approach reveals *Lactobacillus fermentum* as a microbiome-based therapy for COPD

**DOI:** 10.1038/s41522-026-00978-w

**Published:** 2026-04-14

**Authors:** Na Hyun Kim, Jaeik Oh, Jang Ho Lee, Sunyoung Lee, Eun Sung Jung, Dong Ho Suh, Hye-Ji Kang, Bobae Kim, Hye-Shin Kim, Hae Rim Jung, Heeseo Kim, I Na Rae Yun, Yosep Ji, Sung-Yup Cho, Sei Won Lee

**Affiliations:** 1 Department of Pulmonology and Critical Care Medicine, Asan Medical Center, University of Ulsan College of Medicine, Seoul, Republic of Korea; 2 Department of Translational Medicine, Seoul National University College of Medicine, Seoul, Republic of Korea; 3 Medical Research Center, Genomic Medicine Institute, Seoul National University College of Medicine, Seoul, Republic of Korea; 4 HEM Pharma Inc., Suwon, Republic of Korea; 5 Microbiome Convergence Research Center, Korea Research Institute of Bioscience and Biotechnology (KRIBB), Daejeon, Republic of Korea; 6 Department of Biomedical Sciences, Seoul National University College of Medicine, Seoul, Republic of Korea; 7 Cancer Research Institute, Seoul National University, Seoul, Republic of Korea

**Keywords:** Diseases, Medical research, Microbiology

## Abstract

Chronic obstructive pulmonary disease (COPD) remains a major health burden with few effective therapies, particularly for emphysema. The gut–lung axis and microbial metabolites, such as short-chain fatty acids (SCFAs), have emerged as modulators of lung inflammation. We investigated the therapeutic effects of *Lactobacillus fermentum* HEM20792 (LF), identified through a colon mimetic personalized pharmaceutical meta-analytical screening (PMAS) platform using fecal samples from severe COPD patients. LF and *Lactobacillus sakei* HEM20224 (LS) were orally administered to smoke-exposed mice, followed by lung function testing, histopathology, RNA sequencing, single-cell transcriptomics, and fecal microbiome/SCFAs analyses. LF attenuated emphysematous changes, improved compliance, and reduced macrophage and IL-17+ lymphocyte infiltration. Single-cell analysis showed restoration of alveolar macrophages and reduction of pathogenic C1q^+^ macrophages, while transcriptomics revealed normalization of NF-κB and arachidonic acid pathways and attenuation of IL-17– and SPP1-associated signaling. LF also increased fecal SCFAs levels. These findings provide preclinical evidence for LF as a promising microbiome-based therapeutic candidate for COPD.

## Introduction

Chronic obstructive pulmonary disease (COPD) remains one of the leading causes of morbidity and mortality worldwide, posing a significant global health and economic burden^1^. Despite advancements in pharmacological and non-pharmacological therapies, COPD remains largely incurable, with current treatments primarily aimed at symptom relief and slowing disease progression^2^. The persistent unmet need for innovative therapeutic strategies highlights the urgency for novel approaches targeting the underlying pathophysiological mechanisms of COPD. Smoking-induced emphysema, a major phenotype of COPD, is particularly challenging to treat due to its complex inflammatory, oxidative, and tissue-destructive pathways; to date, definitive treatment remains unavailable^3^.

In recent years, the human microbiome has emerged as a promising area of research, offering new therapeutic avenues for various chronic diseases, including respiratory conditions^4^. Dysbiosis, or an imbalance in microbial communities, has been implicated in the pathogenesis of several inflammatory, immune-mediated diseases^5^, including chronic respiratory diseases^6^. The gut–lung axis—a bidirectional communication network between gut microbiota and lung immunity—has garnered significant attention for its role in respiratory health^7^. Gut-derived microbial metabolites, such as short-chain fatty acids (SCFAs), have been shown to modulate immune responses, reduce pulmonary inflammation, and potentially alleviate disease severity in models of chronic respiratory diseases^8–10^.

Among the diverse gut microbial strains, *Lactobacillus*, a group of probiotic bacteria commonly isolated from fermented foods, has been widely studied for its anti-inflammatory, antioxidant, and immunomodulatory properties^11,12^. Known for their safety profile and ability to survive in human gastrointestinal conditions, *Lactobacillus* spp. have shown therapeutic potential in various disease models, including gastrointestinal disorders, metabolic diseases, and respiratory infections^13^. These probiotics exert their effects through multiple mechanisms, such as modulating gut barrier integrity, reducing systemic inflammation, and producing bioactive metabolites like SCFAs^14^. However, the therapeutic efficacy of *Lactobacillus* spp. in smoking-induced emphysema remains underexplored. Exploring the distinct properties of *Lactobacillus* strains could provide valuable insights into their role as potential therapeutic agents for COPD management.

In this study, we employed a personalized pharmaceutical meta-analytical screening (PMAS) platform, utilizing a colon mimetic platform based on fecal samples from patients with severe emphysema, to identify promising *Lactobacillus* strains with therapeutic potential. Through this innovative screening approach, we selected promising *Lactobacillus* strains and evaluated their effects in a disease model. We hypothesized that these *Lactobacillus* strains could ameliorate the progression of smoking-induced emphysema by influencing gut and pulmonary immune responses through the gut–lung axis. To test this hypothesis, we evaluated their therapeutic efficacy in a smoking-exposed emphysema model combined with viral-mimic stimulation, which reproduces key features of emphysema-related lung injury and inflammation^15–18^.

This study aimed to provide mechanistic insights and preclinical evidence for the therapeutic application of selected *Lactobacillus* strains in a smoking-induced emphysema model. Our findings could pave the way for microbiome-based therapeutic strategies for COPD management, addressing an urgent clinical need in chronic, debilitating diseases.

## Results

### PMAS‑based prioritization of candidate Lactobacillus strains

Table 1 presents the baseline characteristics of twelve donors for prioritizing via the PMAS platform. All donors were male. The median smoking amount was 47.5 (interquartile range, IQR 39.8–71.3) pack×years. Median forced expiratory volume in 1 s and diffusing capacity were 37.0% (IQR 25.5–41.3) and 27.5% (IQR 21.3–36.8) of the predicted values, respectively, and consistent with severe airflow limitation and emphysema. The median COPD assessment test score was 16.5 (IQR 12.5–18.3).

**Table. 1:** Demographic characteristics of the feces donors with COPD

	Feces donors for the PMAS platform (*n* = 12)
Male, *n* (%)	12 (100.0%)
Age, years	68.5 [65.3, 72.3]
Body mass index, kg/m^2^	20.0 [19.0, 21.9]
Smoking status, *n* (%)	
Current smoker	1 (8.3%)
Ex-smoker	11 (91.7%)
Smoking amount, pack × years	47.5 [39.8, 71.3]
Pulmonary function test	
FEV_1_ measured, *L*	1.20 [0.72, 1.31]
FEV_1_% predicted	37.0 [25.5, 41.3]
FVC measured, *L*	3.33 [3.22, 4.05]
FVC% predicted	78.5 [74.3, 90.3]
FEV_1_/FVC	31.0 [22.3, 39.0]
DLco, mL/min/mmHg	5.9 [4.7, 7.8]
DLco % predicted	27.5 [21.3, 36.8]
Inhaler use	
LAMA + LABA	8 (66.7%)
LAMA + LABA + ICS	4 (33.3%)
COPD assessment test score	16.5 [12.5, 18.3]
Moderate to severe exacerbation in the last year	0 (0%)

Data are presented as median and interquartile range or number (%).

*COPD* chronic obstructive pulmonary disease, *DLco* carbon monoxide diffusion in the lung; *FEV*_*1*_ forced expiratory volume in 1 s, *FVC* forced vital capacity, *ICS* inhaled corticosteroid, *LABA* long-acting beta-2 agonizts, *LAMA* long-acting muscarinic antagonists.

**Fig. 1:**
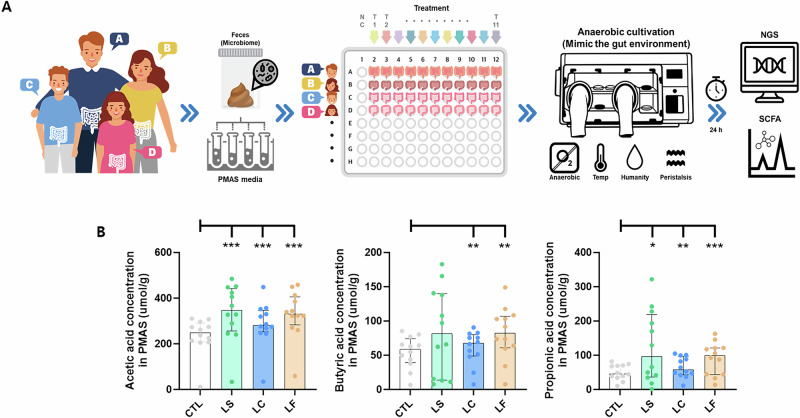
PMAS-based prioritization of therapeutic candidate strains. **A** Process of the personalized pharmaceutical meta-analytical screening (PMAS) platform. Quantified fecal samples from each participant are homogenized and inoculated onto prepared culture media to mimic the gut environment. The filtered supernatant from each participant is subsequently dispensed into the horizontal rows of a 96-well plate. Using the measured CFU values of the desired microbes, an equivalent CFU is distributed in the vertical columns. Following continuous anaerobic incubation at 37 °C for 24 h, a portion of the supernatant is analyzed for SCFAs. **B** Boxplots of SCFAs levels after the PMAS platform experiments. All box and whisker plots illustrate the median and the interquartile range. *P < 0.05 by paired t-test. CFU colony-forming unit, LC *Lactobacillus curvatus* HEM20382, LF *Lactobacillus fermentum* HEM20792, LS *Lactobacillus sakei* HEM20224*,* PMAS personalized pharmaceutical meta-analytical screening, SCFAs short-chain fatty acids.

Utilizing feces from patients with severe COPD in the PMAS platform, *Lactobacillus* strains that produce more SCFAs in the colon mimetic platform were identified (Fig. 1B). Compared to the controls, *Lactobacillus fermentum* HEM20792 (LF) and *Lactobacillus curvatus* HEM20382 (LC) in acetate, propionate, and butyrate showed significant increases in SCFAs concentration; *Lactobacillus sakei* HEM20224 (LS) increases acetate and propionate production, not butyrate. The mean levels of three SCFAs were higher in LF and LS compared to control and LC (Supplementary Table 1). Based on these results, we selected LS and LF as candidate strains for the animal experiment considering both mean concentrations of SCFAs and statistical significance. In a complementary postbiotic experiment, butyrate supplementation in a smoke-exposed model attenuated airspace enlargement and reduced inflammatory markers, supporting the plausibility of an SCFA-linked mechanism (Supplementary Fig. 1), consistent with the previous studies^8^.

### Emphysema development after Lactobacillus strains administration

**Fig. 2:**
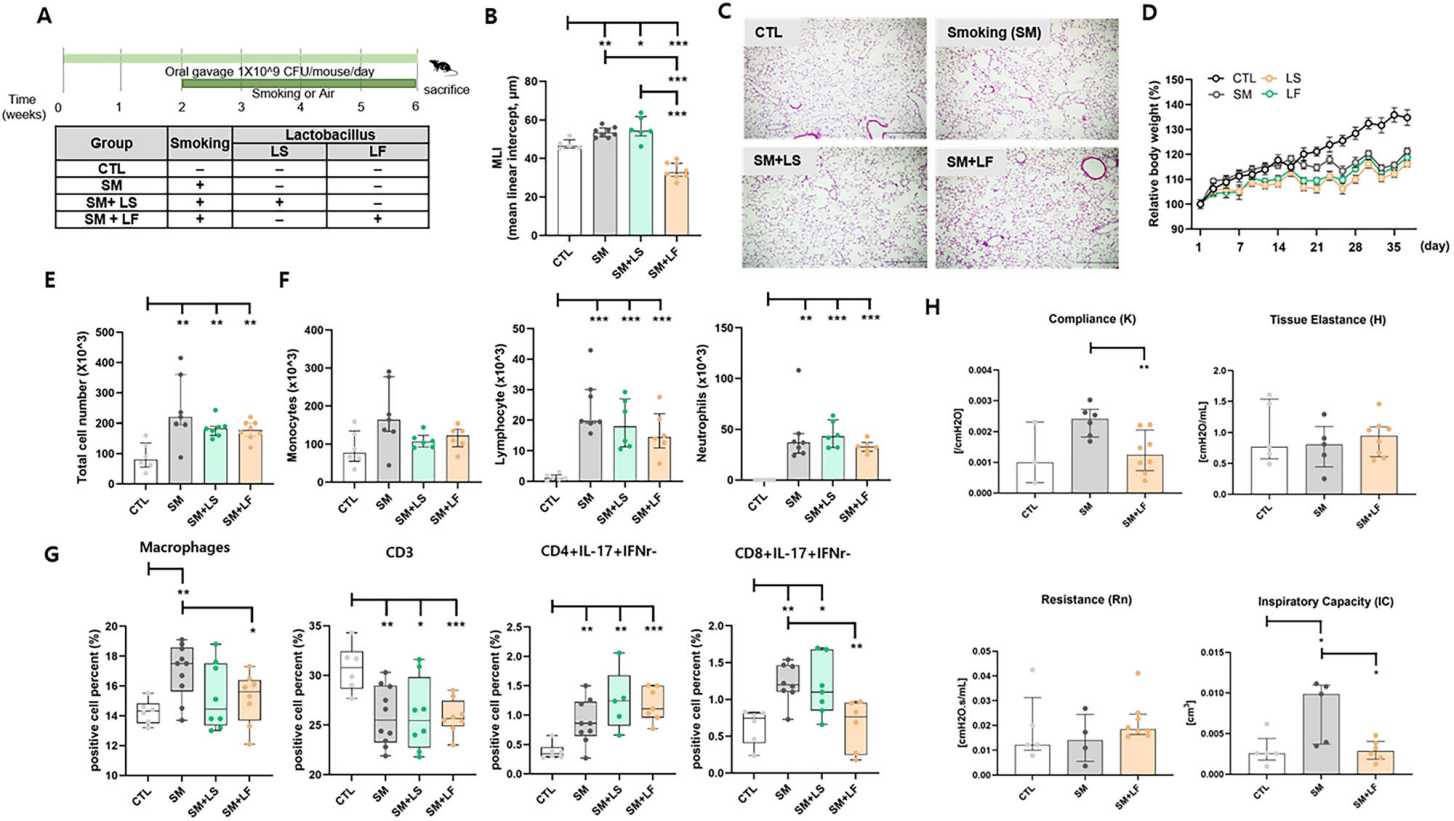
Application of therapeutic candidate strains to the smoking-exposed emphysema model. **A** Experimental design of smoking-exposed emphysema with feeding PBS or *Lactobacillus* strains. **B** Emphysema development is assessed by calculating the MLI of lung tissue. **C** Hematoxylin and eosin staining of the left lung histology. Representative images are shown. Scale bar = 250 µm (magnification: ×100). **D** Body weight change **E** Total number of cells in the bronchoalveolar lavage fluid. **F** Differential cell counts in the BAL fluid. **G** The proportion of macrophage, CD3+ T cells, CD4+ IFN-γ + T cells, and CD8 + IL-17 + T cells in the lung tissues. **H** Pulmonary mechanics assessed by the FlexiVent forced oscillation technique and pressure–volume (PV) loop maneuvers. Shown are respiratory system compliance (K), tissue elastance (H), Newtonian resistance (Rn), and inspiratory capacity (IC), reflecting changes in airway resistance and tissue mechanics. A two-sample t-test was used. All box and whisker plots illustrate the median, interquartile range, and largest and smallest observed values. *P < 0.05, **P < 0.01, and ***P < 0.001. BAL bronchoalveolar lavage, CTL control group, IFN interferon, IL interleukin, LF *Lactobacillus fermentum* HEM20792, LS *Lactobacillus sakei* HEM20224, MLI mean linear intercept, SM smoking, TNF tumor necrosis factor.

Based on the PMAS platform results, the animal experiment was conducted to investigate the impact of LS and LF on emphysema development. The brief experiment process is described in Fig. 2A. The mice group that was administered with LF exhibited attenuated emphysema development as assessed by mean linear index (MLI) (Fig. 2B, C). Body weight trajectories during the exposure period were comparable across groups, with no apparent treatment-related weight loss observed in the LS- or LF-administered mice (Fig. 2D). Inflammatory cell counts in bronchoalveolar lavage fluid (BALF) were increased in the smoking-exposed groups (Fig. 2E). Lymphocyte and neutrophil counts and the BALF and mRNA expression levels of proinflammatory mediators in the lung tissue also presented similar trends (Fig. 2F and Supplementary Fig. 2). The proportion of macrophages and CD8^+^IL-17^+^IFN-γ^−^ cells in the lung was significantly increased in the smoking-only group compared with the control group. However, these increases were reversed in the LF group (Fig. 2G). Pulmonary function testing using the FlexiVent system (SCIREQ, Montreal, Canada) showed that LF administration reversed the smoking-induced impairments in lung compliance and inspiratory capacity (Fig. 2H).

### Gut microbiome and metabolites following Lactobacillus strains administration

**Fig. 3:**
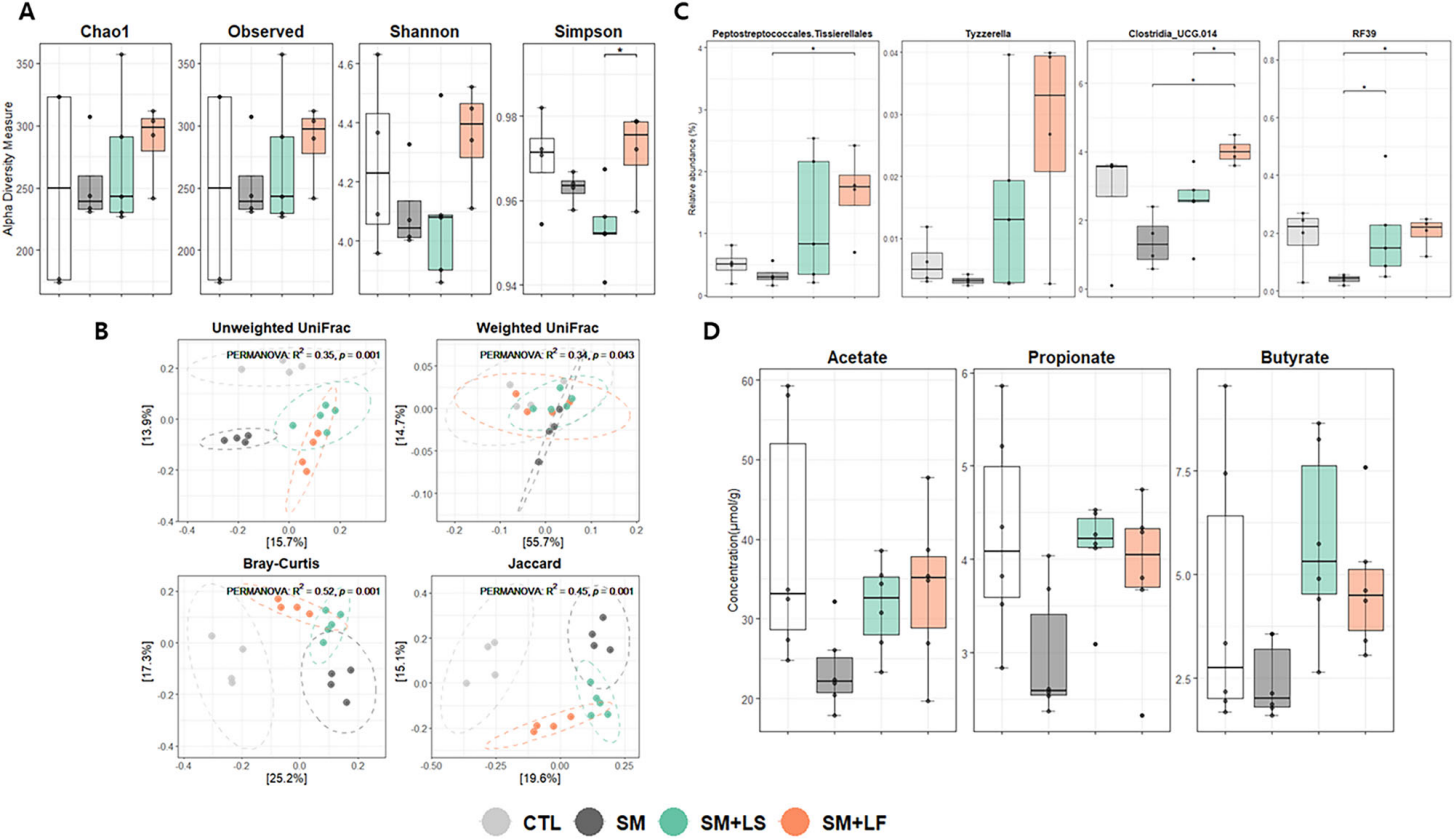
Microbiome analysis of mice feces in smoking-exposed emphysema model. **A** Box plots of alpha-diversity indices (Chao 1, Observed, Shannon, and Simpson Indices). The Wilcoxon rank sum test was used. **B** Beta-diversity indices (PCoA of unweighted UniFrac, weighted UniFrac, Bray–Curtis, and Jaccard Indices). PERMANOVA was performed with 999 permutations; *R*² indicates the proportion of variance explained by the group. **C** Bacterial groups are presented in order of abundance in the LS and LF groups. The Wilcoxon rank sum test was used. **D** Boxplots of short-chain fatty acid levels in the five groups. LF *Lactobacillus fermentum* HEM20792, LS *Lactobacillus sakei* HEM20224.

Mice feces were collected from the colon for microbiome and metabolite analysis. The alpha and beta diversity analyses are presented in Fig. 3A, B. The LF group showed a tendency to increase in each taxonomical analysis compared to the LS group (Fig. 3C). Notably, several taxa (e.g., RF39 and Peptostreptococcales Tissierellales) were consistently identified across multiple differential abundance analyses (Supplementary Fig. 3). Although not statistically significant, the LS and LF groups exhibited a similar pattern of increased acetic acid, propionic acid, and butyric acid levels relative to the smoking-only group. These results concur with the results obtained from the PMAS platform (Fig. 3D).

### RNA sequencing of mice lung tissue following Lactobacillus strain administration

**Fig. 4:**
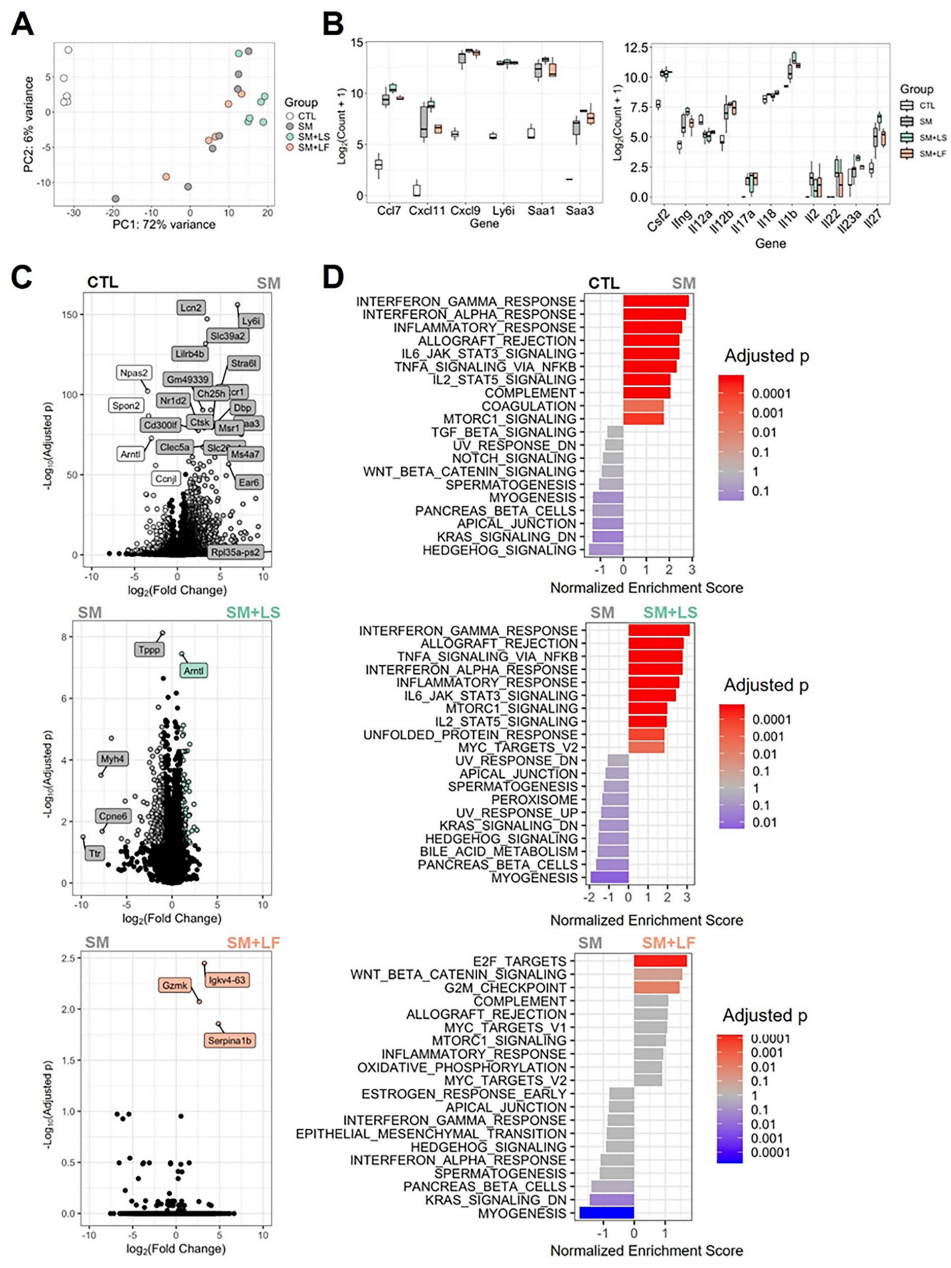
Bulk RNA sequencing analysis on the lung tissues from smoking-induced mouse emphysema models treated with Lactobacillus strains. **A** Principal component analysis (PCA) of bulk RNA sequencing samples. **B** Normalized expression of chemokine and interleukin data across bulk RNA sequencing samples. **C** Volcano plots of the differentially expressed genes between the samples. *P*-values were adjusted for multiple comparisons using the Benjamini–Hochberg false discovery rate (FDR) method. Significant DEGs are marked with colors corresponding to the color of each sample (adjusted *P* value < 0.05 and ∣log2[Fold change]∣ > 0.5). **D** Bar plots of GSEA result derived from hallmark gene set. Log scale of adjusted *P* value which were adjusted for multiple comparisons using the Benjamini-Hochberg FDR method was indicated in colors. CTL control, SM smoking only, SM + LF smoking and *Lactobacillus fermentum* HEM20792, SM + LS smoking and *Lactobacillus sakei* HEM20224.

To elucidate the specific mechanisms underlying the attenuation of the emphysema phenotype in the LF group, we performed bulk RNA sequencing using five mice per group. Principal component analysis (PCA) revealed a distinct separation between the control and smoking-only groups, whereas the LF and LS groups clustered more closely with the smoking-only group (Fig. 4A). RNA expression levels of various cytokines, elevated in the smoking-only group, were not reversed with LF or LS administration (Fig. 4B). The smoking-only group expressed a large number of differentially expressed genes (DEGs) compared to the controls (2066 upregulated and 1853 downregulated genes, adjusted *P* value < 0.05 and ∣log_2_[Fold change]∣ > 0.5). Additionally, the administration of LF or LS resulted in transcriptomic changes in the smoking model. Specifically, LS administration resulted in 338 upregulated and 497 downregulated genes, while LF led to the upregulation of three genes (adjusted *P* value < 0.05 and ∣log_2_[Fold change]∣ > 0.5; Fig. 4C). Gene set enrichment analysis (GSEA) revealed significant upregulation of immune-related gene sets. IFN-γ and IFN-α responses were significant with smoking, and E2F targets were prominent following LF administration in the smoking group (Fig. 4D).

### Lung single-cell analysis following LF administration

**Fig. 5:**
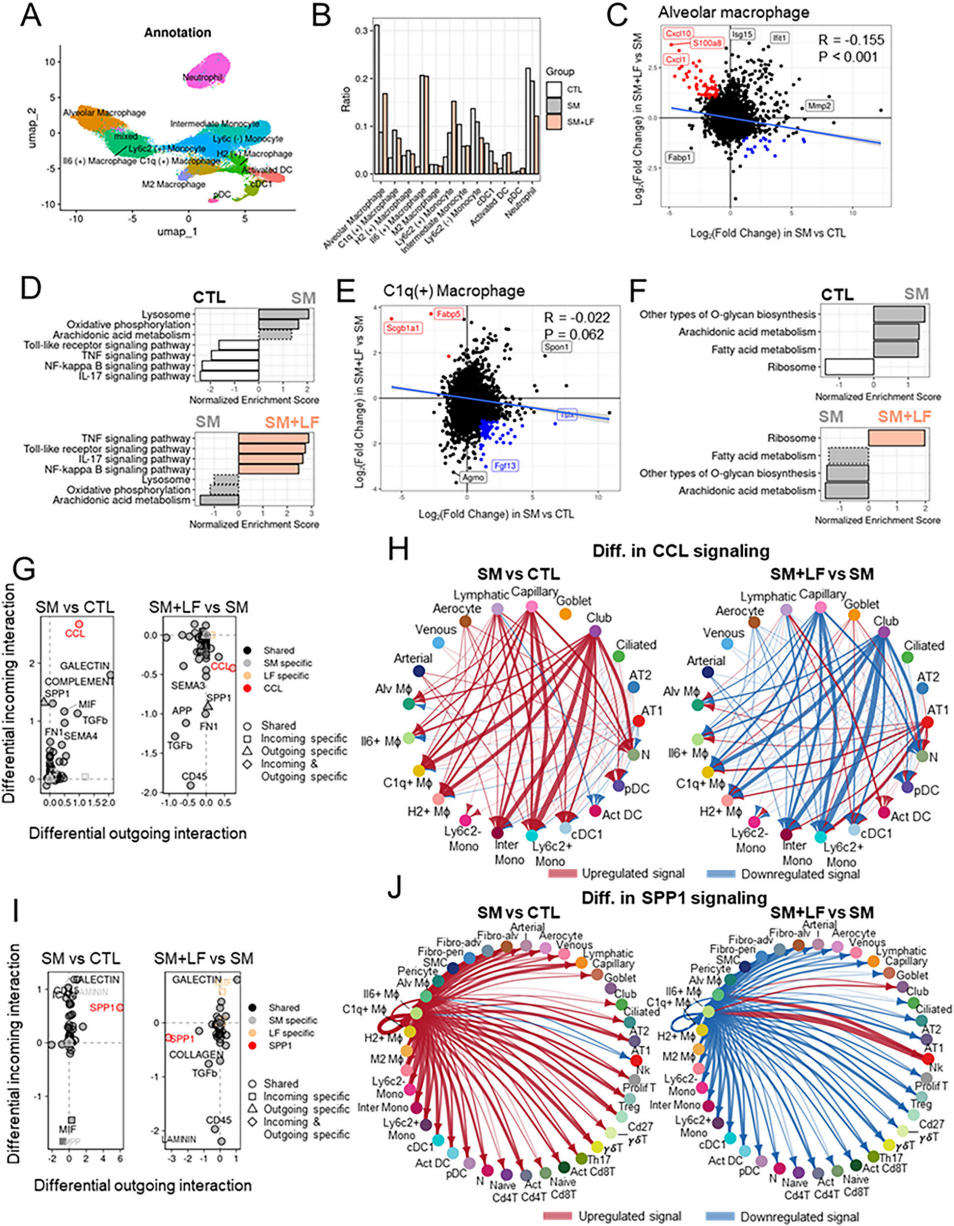
Single-cell sequencing analysis on the lung tissues from smoking-induced mouse emphysema models treated with LF. **A** Uniform Manifold Approximation and Projection (UMAP) representing single cells that were classified as granulocytes. **B** The bar plot displays the proportion of cell types across samples. The *y*-axis of the bar plot indicates the ratio of cell types, while the *x*-axis of the plot represents the types of cells. **C** Comparing differentially expressed genes (DEGs) of the alveolar macrophages between the control and smoking models, with DEGs of the smoking and smoking with LF models. **D** Bar plots of the GSEA result derived from KEGG gene sets. Pathways with plain lines are statistically significant (*q* value < 0.25), while dotted lines indicate non-significant *q* values. **E** Comparing DEGs of C1q^+^ macrophage between the control and smoking models, with DEGs between smoking and smoking with LF model. **F** Bar plots of GSEA results derived from the KEGG gene sets. Pathways with plain lines are statistically significant (*q* value < 0.25), while dotted lines indicate non-significant *q* values. **G** Relative changes in intensity of outgoing or incoming ligand–receptor interactions between C1q^+^ macrophages in each sample. Relative intensity of outgoing ligand–receptor pairs between the smoking and control models (left) and between the smoking with LF and smoking models (right) is plotted on the *x*-axis. Relative intensity of incoming ligand–receptor pairs is plotted on the *y*-axis. **H** Differential strength of intercellular CCL signaling in granulocytes between the smoking and control groups (left) and differential strength between smoking with LF and smoking (right) is plotted using a circular plot. The width of arrows is weighted by their maximal intensity. AT1 alveolar type 1 cells, AT2 alveolar type 2 cells, Ciliated ciliated cells, Club club cells, Goblet goblet cells, Capillary capillary endothelial cells, Lymphatics lymphatic endothelial cells, Venous venous endothelial cells, Arterial arterial endothelial cells, Alv M ϕ alveolar macrophage, Mϕ macrophage, Mono monocytes, cDC1 conventional type 1 dendritic cells, Act DC activated dendiritic cells, pDC plasmacytoid dendiritic cells. **I** Relative changes in intensity of outgoing or incoming ligand–receptor interactions between alveolar macrophages in each sample. Relative intensity of outgoing ligand–receptor pairs between the smoking and control models (left) and between the smoking with LF and smoking models (right) is plotted on the *x*-axis. Relative intensity of incoming ligand–receptor pairs is plotted on the *y*-axis. **J** Differential signaling intensity of outgoing SPP1 from alveolar macrophages and Il6 (+) macrophages. The degree of interaction with SPP1 for each cell type is plotted using the circular plot. The width of arrows is weighted by their maximal intensity. Act Cd4T activated Cd4T, Act Cd8T activated Cd8T, Fibro-alv alveolar fibroblast, Fibro-adv adventitial fibroblast, Fibro-peri peri-bronchial fibroblast, SMC smooth muscle cells, Treg regulatory T cell, Prolif T proliferating T.

Gel beads in emulsion (GEM) were formed with over 40,000 cells per sample. Specifically, 20,157, 21,326, 25,390, and 23,157 cells were recovered for each group (Supplementary Fig. 4a). Cells were annotated into five categories: endothelial cells, epithelial cells, granulocytes, lymphocytes, and stromal cells (Supplementary Fig. 4b). Each group was then further subdivided into subgroups through re-clustering. Single-cell sequencing analysis revealed that, when only granulocytes were clustered separately, two major macrophage populations were identified: residual macrophages, including alveolar macrophages, and recruited macrophages, such as C1q^+^ macrophages (Fig. 5A). Alveolar macrophages are generally recognized for their immunosuppressive and tissue-reparative functions^19,20^. In contrast, recruited macrophages are known to promote immune activation, tissue destruction, and COPD progression^19,20^. Upon macrophage subtyping, smoking exposure led to a decline in alveolar macrophages, while increasing the proportion of C1q^+^ macrophages. Upon LF administration, the alveolar macrophage population was restored, whereas the prevalence of C1q^+^ macrophages was reduced (Fig. 5B). These alterations were evident at the cellular composition and transcriptional levels. Notably, the altered transcriptomic profile of alveolar macrophages caused by smoking was restored following LF treatment (Fig. 5C). GSEA based on DEGs demonstrated that NF-κB signaling in alveolar macrophages was downregulated by smoking but subsequently restored upon LF treatment (Fig. 5D). Similarly, C1q^+^ macrophages exhibited a reversal of smoking-induced DEG patterns following LF administration (Fig. 5E). Furthermore, the upregulation of arachidonic acid metabolism, a key marker of macrophage activation^21,22^, observed in the smoking group was attenuated after LF treatment (Fig. 5F).

To further investigate the mechanism underlying the expansion of C1q^+^ macrophages, cell–cell interaction analyses were conducted using CellChat. The most significantly altered incoming signaling pathway in response to both smoking and LF administration was the CCL–chemokine axis, which plays a pivotal role in macrophage homing within tissues (Fig. 5G). Subgroup analysis revealed that smoking enhanced CCL–CCR interactions between recruited macrophages (including C1q^+^ macrophages) and capillary endothelial cells or certain epithelial cells, such as club cells (Fig. 5H). These interactions were attenuated following LF treatment, suggesting that CCL signaling partially regulates macrophage subtype composition (Fig. 5H). Additionally, previous reports indicated that SPP1 (osteopontin) signaling originating from alveolar macrophages contributes to lung fibrosis and tissue damage^23^. In this study, single-cell transcriptomic analysis confirmed that SPP1 interactions between alveolar macrophages and other types of cells were markedly elevated across most cell types, except alveolar type 1 epithelial cells, in the smoking group (Fig. 5I, J). Notably, LF administration mitigated this aberrant SPP1 signaling, further supporting its potential role in ameliorating smoking-induced pulmonary damage (Fig. 5J). Further analyses for other immune cells, including lymphocytes and NK cells, are available online (Supplementary Figs. 4–6).

To extend our single-cell sequencing analysis beyond immune populations, we examined non-immune cell compartments, including endothelial cells, fibroblasts, and epithelial cells, to evaluate the effects of smoking exposure and LF treatment on tissue remodeling. Transcriptomic alterations in capillary endothelial cells induced by smoking exposure were generally reversed by LF treatment (Supplementary Fig. 6a). GSEA revealed that the IL-17 signaling pathway was specifically enriched in the smoking model and subsequently downregulated following LF treatment (Supplementary Fig. 6b, c). Since not only IL-17 signaling but also Wnt and TGF-β signaling pathways significantly influence fibroblast activation and emphysema formation^24,25^, we evaluated ligand-receptor interactions within the Wnt and TGF-β pathways in alveolar fibroblasts. Consistent with previous findings, transcriptomic changes in alveolar fibroblasts induced by smoking exposure were reversed following LF treatment (Supplementary Fig. 6d). Wnt and TGF-β signaling in alveolar fibroblasts were accentuated in the smoking-only model, while LF treatment attenuated both signals (Supplementary Fig. 6e, f). The ligands responsible for these interactions originated from ciliated epithelial cells (Wnt) and granulocytes (TGF-β), respectively (Supplementary Fig. 6g). In the epithelial cell population, transcriptomic changes induced by smoking exposure were largely reversed by LF treatment (Supplementary Fig. 6h, i), although the relatively small number of epithelial cells limited the detection of differentially expressed genes. Consequently, only a limited number of gene sets showed significant reversal in GSEA beyond immune-related response pathways (Supplementary Fig. 6j).

To determine whether SCFA-related transcriptional programs underlie the immune alterations observed following LF treatment, we identified DEGs from publicly available transcriptomic datasets of murine CD4⁺ T cells (GSE139631 from the Gene Expression Omnibus (GEO) database) and human macrophages exposed to butyrate (GSE248578 from the GEO database; Supplementary Fig. 7a, b). These DEGs were categorized into gene sets that were either positively or negatively regulated by butyrate and subsequently used for GSEA in specific immune cell populations within our single-cell dataset. Despite differences in experimental context and species, GSEA revealed consistent directional enrichment patterns between in vitro butyrate exposure and in vivo LF treatment across multiple macrophage and T cell subsets (Supplementary Fig. 7c). Collectively, these results suggest that butyrate-associated gene programs substantially overlap with immune transcriptomic changes induced by LF, implicating SCFA modulation in LF-mediated immune regulation in COPD.

### Safety analysis of Lactobacillus strains

Supplementary Table 2 presents the safety analysis of LF and LS. Both strains exhibited γ-hemolysis in the hemolysis assay and tested negative for biogenic amine production. Negative results were also observed in the gelatin hydrolysis assay. Additionally, in the antibiotic resistance test, neither strain exceeded the established resistance thresholds.

## Discussion

In this study, the therapeutic potentials of *Lactobacillus* strains for COPD were investigated. Using feces from patients with COPD in the PMAS platform, we identified that LF *and* LS administration demonstrated increased acetate and propionate levels, which are potentially beneficial metabolites, in a colon mimetic platform. In the smoking-exposed emphysema model, LF attenuated the emphysematous change in smoking-exposed mice. Single-cell analysis suggested that the administration of LF affects the inflammatory process, changes the microbiota, and impacts SCFAs levels. In combination, these changed the underlying mechanism of emphysema. This study highlighted the therapeutic potential of LF in COPD, which is still considered a refractory disease.

The development process of microbiome-based therapeutics requires an effective screening and prioritization system to select appropriate strains with therapeutic potential. In this study, we employed the PMAS platform, a colon mimetic platform that used fecal samples from patients with severe emphysema (median FEV₁ 37.0%) to recreate a disease-specific gut environment. This approach allowed us to screen bacterial strains that can produce target metabolites under conditions reflective of the altered microbial and metabolic landscape in patients with severe COPD^8,26^. Traditional methods for probiotic discovery often rely on general probiotic properties and empirical selection of known strains or limited in vitro assays^27^. Based on the beneficial effect of dietary fiber^28,29^ and its metabolites^8^ on COPD, we screened probiotics that produced more SCFAs in the colon mimetic platform of patients with COPD. The strains used in our study were selected among strains that are generally recognized as safe (GRAS) and isolated from probiotic-containing fermented foods. This is also a basic and essential step for the industrial production of probiotic foods^30–32^. Notably, LS and LF exhibited distinct in vivo effects despite both being *Lactobacillus* strains, highlighting strain-specific functional differences and supporting the utility of PMAS platform-based functional prioritization rather than class-level generalizations.

Using the PMAS platform process, we selected two *Lactobacillus* strains. Of these, LF produced significant results. LF attenuated smoking-induced emphysema combined with reduced macrophage infiltration and IL-17-producing lymphocytes in the lung. These findings align with COPD and emphysema pathogenesis^33^, where macrophage^34,35^ and Th17-driven neutrophilic inflammation perpetuate tissue destruction^36–38^. Single-cell analysis further showed that LF reversed smoking-induced shifts in macrophage subtypes, restoring alveolar macrophages while reducing pathogenic C1q^+^ macrophages. Transcriptomic data revealed normalization of NF-κB and arachidonic acid pathways, supporting a role for LF in modulating key inflammatory networks. These effects may partly reflect SCFAs-mediated immunoregulation, including M2 macrophage polarization and attenuation of IL-17 signaling.

Beyond immune modulations, LF also influenced intercellular signaling pathways relevant to emphysema progression. Cell–cell interaction analysis indicated that smoking enhanced CCL–chemokine signaling and SPP1-mediated profibrotic interactions, both of which were attenuated by LF. From a quantitative perspective, the increase in CCL^39^ expression and SPP1^40^ in response to smoking is well-reported, yet evidence on the therapeutic targets is limited. Notably, SPP1 signaling is functionally connected to IL-17 activity^41^, as IL-17 can enhance osteopontin expression, thereby promoting macrophage recruitment, fibrosis, and tissue damage. This synergistic interaction contributes to persistent inflammation and remodeling in COPD. In our model, LF administration reduced both IL-17–associated signaling and aberrant SPP1 interactions, indicating that its effects include the interruption of this pathogenic cytokine–mediator axis. Moreover, smoking-induced upregulation of Wnt and TGF-β pathways in stromal cell signaling networks was reduced after LF treatment, thus implicating additional mechanisms in tissue remodeling. These observations are in line with a previous report implicating epithelial–stromal interactions and aberrant TGF-β signaling in the fibrotic remodeling of lung tissue^42^. Collectively, these findings position LF as a probiotic capable of targeting the immune and structural components of COPD pathology. However, because administration began prior to smoke exposure, our design primarily tests preventive effects. Future studies initiating LF treatment after disease establishment are needed to evaluate its therapeutic potential.

LF is widely isolated from fermented foods, such as kimchi, cheese, and pickles, and has been studied for its safety and antimicrobial and immunomodulatory properties. Different strains of LF suppress inflammatory responses by inhibiting NF-κB and STAT3 signaling, reduce oxidative stress through upregulation of antioxidant enzymes, and protect epithelial barrier integrity^43^. In models of metabolic syndrome, colitis, and respiratory infection, LF administration alleviated inflammation, improved host defense, and enhanced tissue recovery^44–46^. These pleiotropic effects are particularly relevant in COPD, a disease characterized by chronic inflammation, oxidative stress, and recurrent bacterial exacerbations. Our findings extend these observations to smoking-induced emphysema, suggesting that LF may serve as a probiotic candidate that targets multiple pathogenic processes, ranging from macrophage dysfunction to aberrant cytokine–mediator signaling to direct immunomodulation and SCFAs-mediated effects.

SCFAs are central mediators of the gut–lung axis, known to regulate immune balance by promoting M2 macrophage polarization^47^, constraining neutrophilic inflammation^48^, and strengthening epithelial barrier integrity^49^. In our study, LF administration increased fecal acetate, propionate, and butyrate, which paralleled attenuation of emphysema, reduced inflammatory cytokine expression, and partial recovery of lung function. These results are in line with previous animal studies that demonstrated that high-fiber diets or fecal microbiota transplantation alleviated smoke-induced emphysema by enhancing SCFAs production and suppressing apoptosis and inflammation^9,10^. Importantly, clinical data also support these findings. Lower fecal SCFAs levels have been linked to worse lung function and greater disease burden in patients with COPD^8^. In this study, fecal acetate levels were positively associated with lung function (FEV_1_) in smokers with emphysema. Additionally, supplementation with acetate and propionate attenuated smoke-induced emphysema and reduced the production of pro-inflammatory cytokines as well as the population of CD3^+^CD4^+^IL-17^+^ T cells in mice. Taken together, our study underscores the role of SCFAs as a key mechanistic link between gut microbiota modulation and protection against smoking-induced lung injury, reinforcing the therapeutic relevance of targeting microbial metabolites.

Despite these promising findings, this study has several limitations. First, strain selection in this study was primarily based on screening utilizing a colon mimetic platform, which may not capture the full range of relevant microbial functions. Other metabolites and host–microbe interactions may also contribute to the observed effects. Second, although LF administration improved emphysema phenotypes in mice, the precise mechanisms linking gut microbiota changes to lung immune modulation remain incompletely defined. In addition, a non-smoking + probiotics group was not included in the present study design; therefore, subtle effects of LF on baseline lung physiology cannot be fully excluded. Third, extrapolation to human COPD requires caution, as the murine smoking model does not fully replicate the complexity and chronicity of COPD in humans. Accordingly, the functional conclusions of this study primarily reflect changes in airway resistance and tissue mechanics rather than alterations in forced expiratory airflow indices.

In conclusion, our findings demonstrate that LF attenuates smoking-induced emphysema by modulating immune responses, restoring macrophage balance, and enhancing SCFAs production. These results highlight the potential of LF as a microbiome-based therapeutic strategy for COPD management, providing a foundation for future clinical studies.

## Methods

### Study population for feces donors

We utilized fecal samples from twelve donors with COPD, aged over 18 years and with a significant smoking history. Informed consent was obtained from all donors to participate in the study and provide a fecal sample between January 2020 and April 2021. The study was approved by the Institutional Review Board of the Asan Medical Center (no. 2020-0314).

### Personalized pharmaceutical meta-analytical screening (PMAS) platform

The PMAS platform was developed to evaluate strain-specific metabolic outputs in a donor-resolved, gut-mimetic ex vivo culture system. Candidate *Lactobacillus* strains evaluated in PMAS were identified using a stepwise prioritization pipeline. Briefly, an in-house isolate library (*n* = 31) was initially screened in RAW264.7 macrophages to prioritize strains that increased IL-10 production without concomitant induction of tumor necrosis factor (TNF)-α. Seven shortlisted strains were then evaluated in an OVA-induced allergic airway inflammation model. Three strains demonstrating consistent attenuation of airway inflammatory markers (LS, LC, LF) were subsequently advanced to PMAS for functional SCFA assessment in COPD-donor fecal microbiomes (Supplementary Fig. 8)^50^. Figure 1A briefly illustrates the process of the PMAS platform. The PMAS platform imitated the intestinal environment of each person and was utilized to screen candidate bacterial strains. The imitated factors included nutrient, temperature, humidity, and motion. Three candidate *Lactobacillus* strains: LF, LS, and LC, were screened for metabolites with therapeutic potentials^8–10^ through the PMAS platform. Fecal samples from 12 patients with severe COPD were used to establish individual donor microbiome cultures (Table 1). Briefly, frozen fecal aliquots were thawed on ice and homogenized in specialized PMAS media (including L-cysteine hydrochloride, mucin, hemin, resazurin sodium salt, sodium chloride, sodium hydrogen phosphate, and potassium chloride) under anaerobic conditions. The fecal slurry was then filtered to obtain the supernatant and remove large particulates while preserving the microbial community. The donor-specific inocula were distributed into a 96-well plate format, with each row corresponding to one donor microbiome. Candidate Lactobacillus strains (1 × 10^11^ CFU/g)—LF, LS, and LC—were treated with fecal samples. Each donor microbiome was incubated in parallel with each strain, alongside matched donor-specific control wells without added strains. Plates were incubated at 37 °C for 24 h under anaerobic conditions (Whitley A95 Workstation, Don Whitley Scientific, Bradford, UK) to simulate gut microbiome activity in individual fecal samples. Following incubation, cultures were immediately stored at –80 °C to halt further reactions until subsequent analysis. Upon thawing, 100 μL of dH2O was added to the samples, and 100 μL of each sample was transferred to a new microcentrifuge tube for SCFA analysis. For further details regarding the PMAS technology, please refer to the related patents (U.S. Patent No. 11,237,172; KR Patent Nos. 10-2124474 and 10-2227382). SCFA quantification: Acetate, propionate, and butyrate were quantified from culture supernatants using gas chromatography with flame ionization detection (GC-FID), employing an internal standard and external calibration curves. SCFA concentrations were reported as µmol/g.

Regarding data processing and statistics, SCFA induction by each strain was evaluated relative to the matched donor control condition using a paired design for each donor. Group-level differences across donors were assessed using a paired t-test.

### Murine model of emphysema

All animal care and experimental procedures were approved by the Institutional Animal Care and Use Committee of the Asan Medical Center (no. 2022-13-126). Eight-week-old female SPF C57BL/6 mice were obtained from Orient Bio Inc. (Seongnam, Republic of Korea). The mice were assigned into four groups: control (*n* = 6), smoking only (*n* = 7–10), LF (*n* = 10), and LS (*n* = 10) treatment groups. Sample sizes were determined based on prior experience with the smoke-exposure model and feasibility considerations; formal a priori power calculations were not performed. Mice were exposed to cigarette smoke in a whole-body apparatus 5 days a week for 4 weeks, using 12 commercial cigarettes per day (four cigarettes/session, three sessions/day, 8.0 mg tar/cigarette, and 0.70 mg nicotine/cigarette; Camel, R. J. Reynolds Tobacco Company, Winston-Salem, NC, USA). Mice in the LF and LS groups were orally administered the respective *Lactobacillus* strain daily from 2 weeks before the start of smoke exposure to one day before sacrifice. During the 4-week smoking exposure period, polyinosinic:polycytidylic acid (Poly (I:C)) was intranasally administered at a dose of 10 mg/kg in a volume of 50 μL/mouse, twice a week in the third and fourth weeks. Poly(I:C) was used as a viral dsRNA mimic to model an exacerbation-like innate immune response during cigarette smoke exposure. All smoke-exposed groups received Poly(I:C), while control mice were exposed to room air only^8,17,18^. The control group inhaled only clean-room air. The experiments were completed in 6 weeks, and the mice were sacrificed subsequently.

### Collection and preparation of mouse samples

At the end of the experimental period, all mice were anesthetized using isoflurane inhalation and euthanized, and blood samples were collected by cardiac puncture. To collect BALF, the trachea was catheterized and injected with 1.5 mL PBS. To separate supernatants and cellular components, the BALF was centrifuged at 2200 rpm for 5 min at 4 °C. The cell pellet was resuspended in PBS, placed on a slide, and stained with Diff-Quick (Sysmex, Kobe, Japan). After BALF collection, the lungs were harvested for histology. After ligating the right main bronchus, the left lung lobe was inflated with 0.5% low-melting point agarose at a constant pressure of 15 cmH_2_O and fixed in 10% formalin. The remaining right lobe was collected, snap frozen, and stored at −80 °C for further analysis. The right postcaval lobe was utilized for quantitative real-time PCR analysis.

### Histopathological and mean linear intercept assessment

After fixation, each lung lobe was separated, embedded in paraffin, and cut into 5-µm sections. These sections were stained with hematoxylin and eosin to allow measurement of the MLI and to determine the severity of emphysematous changes. To assess MLI, the mean inter-alveolar septal wall distance was calculated by the number of interruptions in the 1-mm lines of the alveolar wall. Four lines were drawn in each field, and at least five random fields per mouse were examined.

### Measurement of cytokine levels

The levels of TNF-α, IL-6, IL-1β, IL-17A, and IL-18 in serum and BALF samples were measured using a commercially available enzyme-linked immunosorbent assay kit (R&D Systems, Minneapolis, MN, USA) according to the manufacturer’s instructions.

### Quantitative real-time PCR

Total RNA was extracted from lung tissue using TRIzol reagent (Thermo Fisher Scientific, Waltham, MA, USA). Total RNA (1 μg) was used to synthesize cDNA using the primeScript RT Master Mix kit (Takara Bio Inc., Kyoto, Japan). Transcripts were quantified using real-time PCR using sequence-specific primers for TNF-α, IL-6, IFN-γ, and IL-1β. Amplification reactions were performed using the Advanced Universal SYBR Green Supermix kit and the CFX Connect Real-Time PCR system (Bio-Rad Laboratories, Hercules, CA, USA). Target gene expression levels were normalized to 18S RNA as an endogenous control gene. The 2^−ΔΔCT^ method was used to calculate the relative changes.

### Pulmonary function test in smoking-exposed emphysema model

To evaluate lung function in smoking-exposed mice, pulmonary mechanics were assessed using the FlexiVent FX system (SCIREQ, Montreal, Canada). Mice were anesthetized with an intraperitoneal injection of Zoletil (tileamine hydrochloride and zolazepam, 50 mg/mouse), followed by tracheostomy and the insertion of an 18-gauge metal cannula. The animals were then connected to the FlexiVent ventilator and ventilated at a tidal volume of 10 mL/kg, a respiratory rate of 150 breaths/min, and a positive end-expiratory pressure of 3 cmH₂O. After stabilization, pulmonary mechanics were measured using the forced oscillation technique and pressure–volume (PV) loop maneuvers. Forced-expiration maneuvers were not performed because the dedicated FlexiVent module/software for these measurements was not available in our setup. The following parameters were measured: respiratory system resistance (Rrs, total airway resistance), Newtonian resistance (Rn, resistance of the central conducting airways, tissue elastance (H, a measure of tissue stiffness and peripheral airway resistance), and lung volume (A, assessed as the area under the PV loop, representing lung capacity). Each perturbation was performed in triplicate or quadruplicate and averaged for the analysis. PV loops were obtained by slowly inflating and deflating the lungs from 0 to 30 cmH₂O under quasi-static conditions. All measurements were analyzed using FlexiWare (version 8.3, SCIREQ). Outliers and artifacts were excluded based on quality control criteria, including a coefficient of determination (*R*²) threshold of ≥0.95.

### Measurement of short-chain fatty acids

All SCFAs were extracted from 0.2 g of fecal samples in 1 mL of dH_2_O. Homogenized samples were centrifuged at 13,000 rpm for 10 min at 4 °C. Following centrifugation, the supernatant (150 μL) was transferred to a 10 mL screw cap vial with 150 μL of GC buffer solution containing (NH_4_)_2_SO_4_ and NaH_2_PO_4_, and 2-ethylbutric acid was added as internal standard^51^.

Headspace autosampler-gas chromatography-flame ionization detector (HSS-GC-FID) analysis was performed using the Agilent 7890B GC system, equipped with a 7697A headspace sampler and FID (Agilent Technologies, USA). An HP-Innowax capillary column (30 m × 0.32 mm i.d. × 0.50 μL film thickness; Agilent) was used with a constant flow of nitrogen as the carrier gas. The operating conditions were as follows: oven temperature, 85 °C; loop temperature, 90 °C; transfer lines, 100 °C; FID temperature, 250 °C; column temperature was initially at 60 °C, raised to 140 °C at 30 °C/min, then raised to 170 °C at 30 °C, and finally to 180 °C at 40 °C, and held for 0.75 min. Data acquisition and operation processing were conducted using ChemStation software (Agilent Technologies). SCFAs were identified and quantified using standard compounds.

### DNA extraction and metagenomic analysis

DNA was extracted from the fecal samples using Mag-Bind® Universal Pathogen Kit (Omega Bio-tek, Norcross, GA, US). The fecal samples were suspended in 275 μL of SLX-Mlus Buffer, followed by bead beating using Mixermill MM400 (Retsch, Haan, Nordrhein-Westfalen, Germany) with further isolating, cleaning, and eluting procedures, following the manufacturer’s protocols.

PCR was performed using these fecal samples amplicon primers, 2× KAPA HiFi HotStart ReadyMix (Roche, Basel, Basel-Stadt, Switzerland), and DNA under conditions of 3 min at 95 °C, followed by 25 cycles at 95 °C for 30 s, annealing at 55 °C for 30 s, extension at 72 °C for 30 s, and final extension at 72 °C for 5 min. Subsequently, the sample DNA was cleaned with HiAccuBead (AccuGene, Incheon, South Korea) and a magnetic stand. The index PCR was performed using the IDT indexing primer (Integrated DNA Technologies, Coralville, Iowa, US) for the Illumina MiSeq System, 2× KAPA HiFi HotStart ReadyMix, and PCR-grade water. PCR was performed at 95 °C for 3 min (eight cycles of 95 °C for 30 s, 55 °C for 30 s, 72 °C for 30 s, and 72 °C for 5 min) and held at 4 °C for the PCR reaction. After the clean-up step, the concentration of libraries was verified using Qubit 4.0 (ThermoFisher Scientific, Waltham, Massachusetts, US) with 1× dsDNA HS assay solution (ThermoFisher Scientific, Waltham, Massachusetts, US) and sequenced using the Illumina MiSeq system.

Reads were sorted using the unique barcodes for each PCR product. The barcode, linker, and primer sequences were removed from the original sequencing reads. The sequencing results were analyzed using the Qiime2 bioinformatics pipeline. Furthermore, taxonomic assignment was performed using the Silva 138 database.

To investigate diversity, we calculated alpha diversity (the Chao1, Observed, Shannon, and Simpson Indexes) and beta diversity (Principal Coordinate Analysis plot on unweighted UniFrac, weighted UniFrac, Bray–Curtis, and Jaccard). The difference in bacterial taxa level among the groups was assessed by linear discriminant analysis (LDA) effect size (LEfSe), DESeq2 analyses and MaAsLin3 analyses. These steps were performed using an R package. In the LEfSe analysis, genera with an LDA value > 2 and *P* < 0.05 were regarded as significant. In the DESeq2 analysis, the bacterial groups with more than 2 log_2_ fold change and *P* < 0.05 were regarded as significant. For MaAsLin3 analysis, differential abundance testing was performed for microbial taxa from phylum to genus level, excluding uncultured taxa. Relative abundances were normalized using total-sum scaling (TSS) followed by log transformation. No additional covariates were included due to the lack of available metadata beyond group assignment. Taxa with a nominal *P* < 0.05 were included for visualization.

### Bulk RNA sequencing

A cDNA library was prepared with the SureSelectXT RNA Direct kit (Agilent Technologies). Paired-end sequencing was performed on the Illumina platform. Generated FASTQ files were further aligned according to GRCm38.v45 primary assembly genome files downloaded from GENCODE using the STAR aligner via Nextflow rnaseq (23.10.0). The read count of each gene was calculated using RSEM. Raw p-values were corrected for multiple testing using the Benjamini–Hochberg false discovery rate (FDR) approach. Further analysis was performed using the DESeq2 package version 1.42.1 in R. Differentially expressed genes were revealed using non-normalized count data from RSEM. Gene set enrichment analysis was performed with fgsea version 1.28.0 and msigdbr version 7.5.1 packages in R. Differential expression was analyzed with DESeq2, and gene set enrichment was performed using fgsea (v1.28.0) and msigdbr (v7.5.1) packages in R.

### Single-cell RNA sequencing and data analysis

Single-cell suspensions were obtained by chopping the right lungs of PBS-perfused mice and digesting with digesting media (RPMI containing 1 mg/mL Collagenase D [Roche] and 20 U/mL of DNAase I [Qiagen]) at 37 °C for 45 min. The digested lung tissue was passed through a 40-μm cell strainer using a plunger and resuspended in 20 mL of ice-cold RPMI supplemented with 10% FBS. RBC lysis buffer was used to remove red blood cells. Single-cell barcoding and complementary DNA (cDNA) library preparation were performed according to the manufacturer’s protocol (Chromium Next GEM Single Cell 3ʹ Reagent Kits v3.1, dual index).

Each liquid (cell suspensions, beads, master mix, and oil) was loaded on the Chromium Next Gem chip G to generate gel beads in emulsions and recover approximately 10,000 cells per tube. Reverse transcription was performed at 53 °C for 45 min, followed by purification using the Dynabeads cleanup mix. cDNA was amplified for 11 cycles using a thermocycler. cDNA size selection was performed with SpriSelect beads (Beckman Coulter, USA), and cDNA quality was confirmed with an Agilent Bioanalyzer High Sensitivity DNA chip. DNA fragmentation, end-repair, A-tailing, and ligation of sequencing adapters were performed according to the manufacturer’s protocol (10× Genomics, USA). Illumina Novaseq6000 was used to sequence cDNA libraries (aiming for 100 million reads per library). Base call files were demultiplexed into FASTQ files using bcl2fastq2 (Version 2.20). FASTQ files were aligned to the mouse reference genome GRCm38, supported by 10x Genomics.

Downstream analyses were conducted with the Seurat package (v.5.0.1) in R. Low-quality cells (>5% mitochondrial reads, <200 or >6000 features) were excluded, and expression values were normalized, log-transformed, and scaled with regression for mitochondrial content and cell cycle scores. Clustering was performed with the Louvain algorithm, marker genes for each cell type were identified by applying the Seurat FindAllMarkers, and differential expression and enrichment analyses were conducted using the RunDEtest and RunGSEA functions in the SCP library (v.0.5.6). Cell–cell communication was assessed with CellChat (v 1.6.1).

### Single-cell clustering, differential cell expression, and cell population composition analysis

Further analysis was performed using the Seurat package (v.5.0.1) in R. We adopted SoupX (v.1.6.2) and the DoubletFinder (v.2.0.4) library to remove ambient RNA from soup and doublets, respectively. To retain only high-quality cells, those with >5% mitochondrial gene content, <200 detected features, or >6000 features were excluded from the analysis. Expression values were normalized to 10,000 transcripts per cell and log-transformed using a pseudocount of 1. During data scaling, regression was performed to account for mitochondrial gene percentage and cell cycle scores, the latter calculated based on a predefined list of cell cycle markers, from Tirosh et al.^52^.

Louvain clustering was used to group cells, and the cell type clusters were identified using canonical marker genes. Overall marker genes for each cell type were identified by applying the Seurat FindAllMarkers implementation of the Wilcoxon rank-sum test. Cells annotated as “Mixed” were removed prior to downstream analyses. Differentially expressed gene and gene set enrichment analyses between COPD and control cells were performed using the RunDEtest and RunGSEA functions in the SCP library (v.0.5.6), with statistical testing and adjustment for multiple comparison testing performed as described in the “Methods” and Figure legend.

Interactions between cells were analyzed with the Cellchat library (v 1.6.1)^53^. Each sample was analyzed based on annotations with the default ligand receptor interaction database (CellchatDB.mouse). For differential interaction analysis, three samples were integrated into one Cellchat object and visualized with a weighted circle plot, heatmap, and bubble plot.

### Pilot prebiotic add-on experiment

A pilot prebiotic add-on study was conducted to evaluate whether prebiotics provide additional benefit beyond LF at the selected working dose. Smoke-exposed mice received LF (1 × 10⁹ CFU/mouse/day) by oral gavage for 6 weeks. In add-on groups, inulin (INU), galactooligosaccharide (GOS), or fructooligosaccharide (FOS) was co-administered by oral gavage at 20 mg in 200 µL per mouse daily for 6 weeks. CTL and SM groups received matched vehicle gavage. Endpoints included BAL total cell counts, representative lung histology, and lung inflammatory cytokine transcripts by qPCR.

### Butyrate supplementation experiment in a smoke-exposed emphysema model

To provide complementary postbiotic evidence supporting the role of SCFAs, we performed an independent butyrate supplementation experiment in a smoke-exposed emphysema model (Supplementary Fig. 1). Female C57BL/6 mice aged 8 weeks were randomly assigned to three groups: room air control (CTL, *n* = 6), cigarette smoke exposure (SM, *n* = 9), and cigarette smoke exposure with sodium butyrate treatment (SM+butyrate, *n* = 9). Mice in the SM and SM+butyrate groups were exposed to cigarette smoke for 4 weeks under the same conditions as described for the main emphysema model (four cigarettes/session, three sessions/day, 5 days/week).

Sodium butyrate was administered to the SM+butyrate group via drinking water at 200 mM, provided ad libitum and refreshed every 3 days. In addition, sodium butyrate was administered by intraperitoneal injection at 1 g/kg, 2 times per week, according to the schedule shown in Supplementary Fig. 1a. CTL and SM mice received matched vehicle drinking water and intraperitoneal injections of PBS on the same schedule.

At the endpoint, BAL was collected for total cell counts. Lung tissues were harvested for histology and gene expression analyses. Emphysema severity was assessed by H&E staining and quantified using MLI measurements. Lung mRNA expression of inflammatory mediators and butyrate-related markers was quantified by qPCR and expressed as fold change relative to CTL. Statistical analyses were performed as described in the Statistical Analysis section; significance thresholds are indicated in the figure legend.

### Statistical analysis

Clinical data are presented as mean ± standard deviation or median (IQR) for continuous variables and number (percentage) for categorical variables. The two-sample or paired *t*-test was performed using GraphPad Prism statistical software, version 9.0 (GraphPad, La Jolla, CA). *P-*values < 0.05 indicated statistical significance.

## Supplementary information

LF Supplemental.file.20260225.clean.

## Data Availability

The bulk RNA sequencing and single-cell RNA sequencing data to the European Nucleotide Archive (https://www.ebi.ac.uk/ena, Accession numbers: Bulk RNA-seq, PRJEB102132; Single-cell RNA-seq, PRJEB102131).
