# Supplementary material for: A colon mimetic screening approach reveals Lactobacillus fermentum as a microbiome-based therapy for COPD
Source: NPJ Biofilms Microbiomes. 2026 Apr 14;12:117. doi: 10.1038/s41522-026-00978-w (PMC13265733; doi:10.1038/s41522-026-00978-w)
Supplement: Supplementary file 1 — LF Supplemental.file.20260225.clean. [file 41522_2026_978_MOESM1_ESM.docx]

**Supplementary Information**

**Supplementary Results**

***Pilot dose-ranging synbiotic experiment***

To inform LF dose selection, we performed a preliminary dose-ranging synbiotic experiment using a smoke-exposed emphysema model (CTL, SM, SM+INU+LF 10^8^, SM+ INU +LF 10^9^, and SM+INU+LF 10^10^ CFU/day). Among the doses tested, LF 10^9^ CFU/day demonstrated the most favorable profile, including the lowest BAL cellularity and the most consistent reduction in lung inflammatory cytokine levels (e.g., Tnfα, Il6, Ifng, Il1b) compared with LF 10^8^ or 10^10^. The response pattern was non-monotonic across the tested range, and 1×10⁹ CFU/day showed the most consistent anti‑inflammatory signal under these synbiotic (LF+INU) pilot conditions; therefore, we carried forward 1×10⁹ CFU/mouse/day as a practical working dose for subsequent studies. (**Supplementary Figure 9**).

***Pilot Evaluation of Inulin Add-On to LF in Smoke-Exposed Emphysema***

To evaluate whether prebiotics provide an additive benefit beyond LF, we conducted a second pilot experiment comparing LF at 1×10⁹ CFU/mouse/day alone versus LF combined with different prebiotics (CTL, SM, SM+LF 10⁹, SM+LF 10⁹+INU, SM+LF 10⁹+GOS, and SM+LF 10⁹+FOS). Across the assessed endpoints—including BAL cellularity, representative lung histology, and lung inflammatory cytokine measurements—adding INU, GOS, or FOS did not produce a consistent additional improvement beyond LF alone. These pilot results supported proceeding with LF monotherapy in the main study (Figure 2 and Supplementary Figure 10).

***RNA and protein levels of inflammatory cytokines in BALF and lung samples following Lactobacillus strains administration***

**Supplementary Figure 2** presents the cytokine levels of BALF in mice. When compared with those in the smoking-only group, TNFα, IL-6, IL-1β, IL-17A, and IL-18 levels were increased in the LS group (**Supplementary Figure 2a**). mRNA expression levels of the proinflammatory mediators, including TNFα, IL-6, IFN-γ, and IL-1β, were elevated in mice exposed to smoking compared with mice in the control group (**Supplementary Figure 2b**).

***Lung single-cell analysis following L. fermentum HEM20792 administration***

Ten major lymphocyte populations were identified after T cells and NK cells were separately clustered (**Supplementary Figure 4c and d**). Transcriptomic shifts in Cd4T (naive and activated Cd4T) and Th17 cells by smoking exposure were generally reversed by administration of LF (**Supplementary Figure 4e**). Among the Cd4T interactions, MHC-II signaling exhibited the most prominent increase following smoking exposure, which was subsequently decreased after LF treatment (**Supplementary Figure 4f-h**). Antigen presentation via MHC-II between antigen-presenting cells (APCs) and Cd4T is known to activate the JAK-STAT pathway in T cells. In our analysis, we observed mild but consistent changes in JAK-STAT signaling that aligned with the dynamics of MHC-II interactions (**Supplementary Figure 4i and j**). Similar patterns were also observed within the Th17 subset, where MHC-II signaling increased with smoking and decreased after LF administration (**Supplementary Figure 5a and b**). Although not statistically significant, corresponding changes in the JAK-STAT pathway were also detected in Th17 cells (**Supplementary Figure 5c and d**). IL-17, induced by Th17 activation, promotes fibrosis via stromal cell activation in COPD as well as autoimmune diseases, while also contributing to inflammation by facilitating immune cell recruitment through the endothelial cells ^1,2^. The significantly upregulated IL-17a–IL-17r signaling in the smoking model in various cell types, such as ciliated cells, fibroblasts, pericytes, and AT2, was moderately downregulated following LF in the smoking model (**Supplementary Figure 5e and f**).

**Supplementary Tables**

| SCFA, umol/g | Control | *L. sakei*  HEM20224 | *L. curvatus* HEM20382 | *L. fermentum* HEM20792 |
| --- | --- | --- | --- | --- |
| Acetate | 250.1 [208.8, 291.1] | 347.7 [256.4, 441.9] | 281.5 [252.7, 346.7] | 331.8 [283.6, 405.9] |
| Propionate | 46.5 [35.7, 70.0] | 97.4 [36.6, 219.3] | 58.5 [43.2, 96.7] | 99.1 [44.0, 121.2] |
| Butyrate | 59.0 [39.4, 74.2] | 81.5 [13.8, 139.9] | 67.8 [48.9, 80.0] | 82.7 [60.9, 107.2] |

**Supplementary Table 1.** Concentrations of SCFAs in the personalized pharmaceutical meta-analytical screening (PMAS) ~~system~~ platform

Abbreviation: SCFA, short-chain fatty acid.

**Supplementary Table 2. Safety profiles of the candidate *Lactobacillus* strains.**

| **Test** | **Hemolysis** | **Biogenic amine production** | | | | **Gelatine hydrolysis** | **Antibiotics resistance** | | | | | | | | |
| --- | --- | --- | --- | --- | --- | --- | --- | --- | --- | --- | --- | --- | --- | --- | --- |
| Subcategory | Hemolysis activity | Histamine | Cadaverine | Tyramine | Putrescine | Gelatine activity | AMP | CHL | CLI | ERY | GEN | STR | TET | VAN | KAN |
| *L. sakei* HEM20224 | γ | Neg | Neg | Neg | Neg | Neg | 0.5 | 4 | <0.25 | <0.25 | 4 | 32 | 4 | 64 | 16 |
| *L. fermentum* HEM20792 | γ | Neg | Neg | Neg | Neg | Neg | 0.5 | 4 | <0.25 | <0.25 | 2 | 8 | 4 | N/R | 64 |
| *Lactobacillus* facultative heterofermentative  cut-off value | N/A | | | | | | 4 | 4 | 4 | 1 | 16 | 64 | 8 | N/R | 64 |
| *Lactobacillus* obligate heterofermentative  cut-off value |  |  |  |  |  |  | 2 | 4 | 4 | 1 | 16 | 64 | 8 | N/R | 64 |

Abbreviations: AMP, ampicillin; CHL, chloramphenicol; CLI, clindamycin; ERY, erythromycin; GEN, gentamicin; STR, streptomycin; TET, tetracycline; VAN, vancomycin; KAN, kanamycin.

**Supplementary Figures and Legends**

**
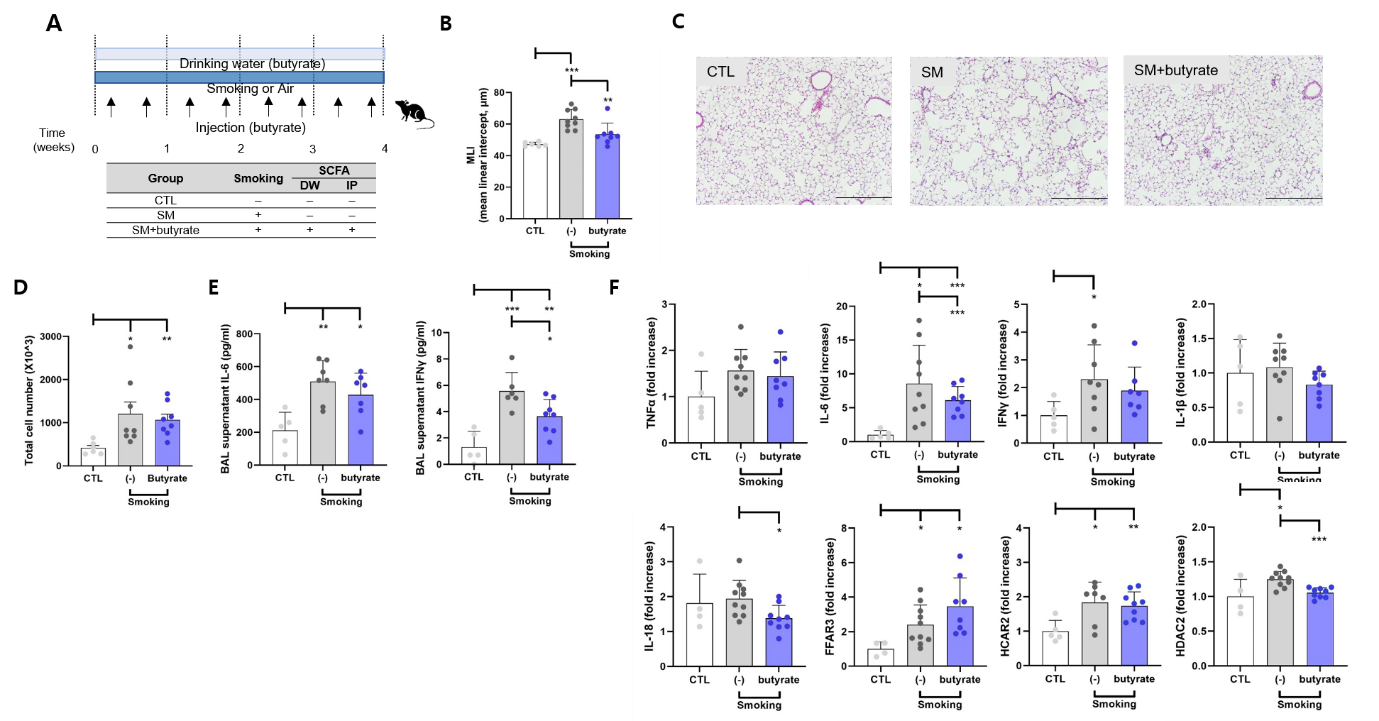
**

**Supplementary Figure 1. Butyrate supplementation attenuates airspace enlargement and inflammatory markers in a smoke-exposed emphysema model.**(A) Experimental scheme: Mice were exposed to cigarette smoke (SM) or room air (CTL) for 4 weeks. Butyrate was administered to smoke-exposed mice via drinking water and intraperitoneal injections according to the schedule shown. (B) Mean linear intercept (MLI). (C) Representative H&E-stained lung sections. (D) Total bronchoalveolar lavage (BAL) cell counts. (E) BAL supernatant cytokine levels (IL-6, IFN-γ). (F) Lung mRNA expression (qPCR; fold change relative to CTL) of inflammatory mediators (TNF-α, IL-6, IFN-γ, IL-1β, IL-18) and butyrate-related signaling/epigenetic markers (Ffar3, Hcar2, Hdac2).
Data are presented as mean values and SDs. Each dot represents one mouse. n = 6 (CTL), 9 (SM), 9 (SM+butyrate). *P < 0.05, **P < 0.01, ***P < 0.001.

**
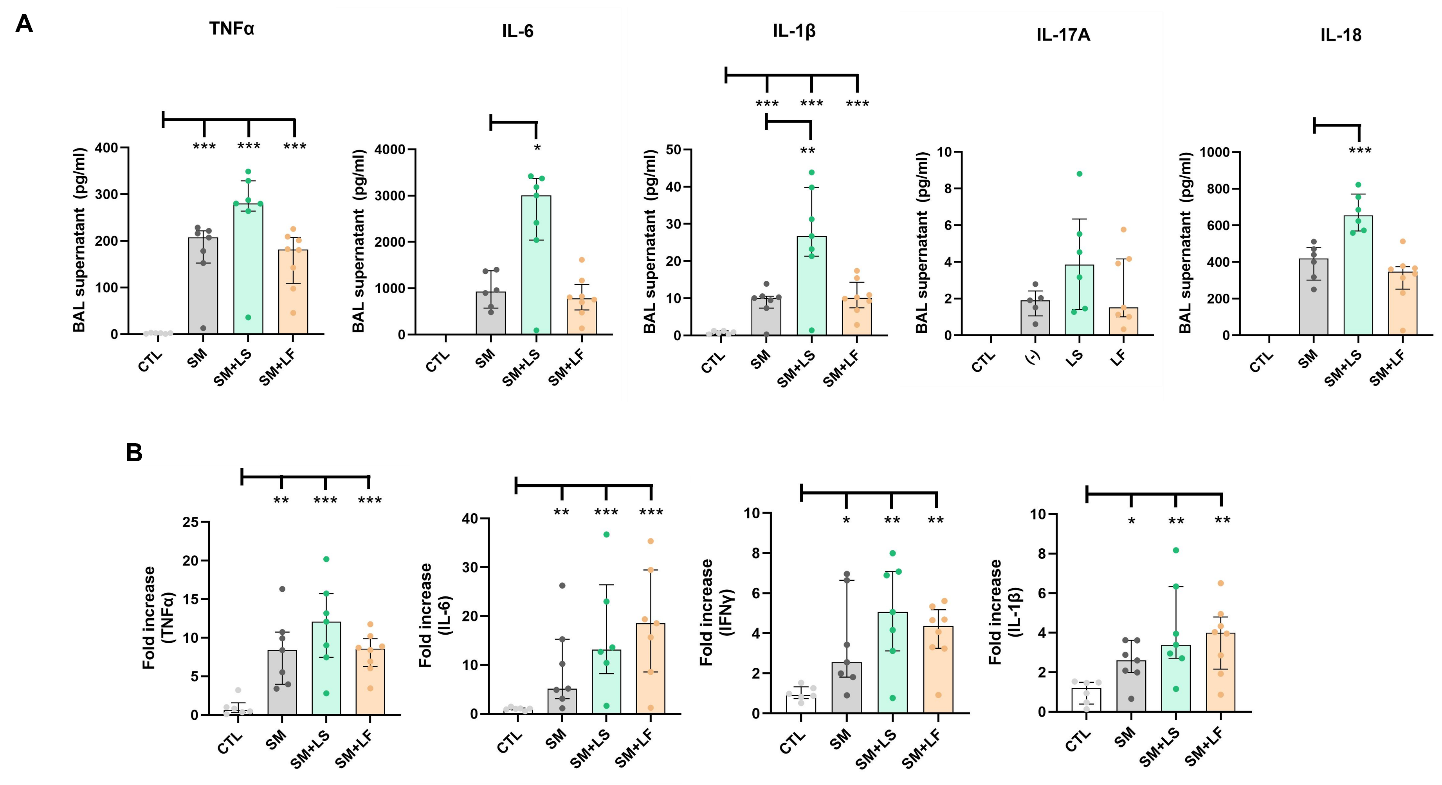
**

**Supplementary Figure 2.** **Cytokine levels in the smoking-exposed emphysema model.**

(a) Levels of TNF-α, IL-6, IFN-γ, IL-1β, IL-17A, and IL-18 in BALF of mice. **(b)** mRNA levels of TNF-α, IL-6, IFNγ, and IL-1β in mouse lung tissue. A two-sample *t*-test was used. Data are presented as mean values and SDs. All box and whisker plots illustrate the median, interquartile range, and largest and smallest observed values. **P* < 0.05, ***P* < 0.01, and ****P* < 0.001.

**
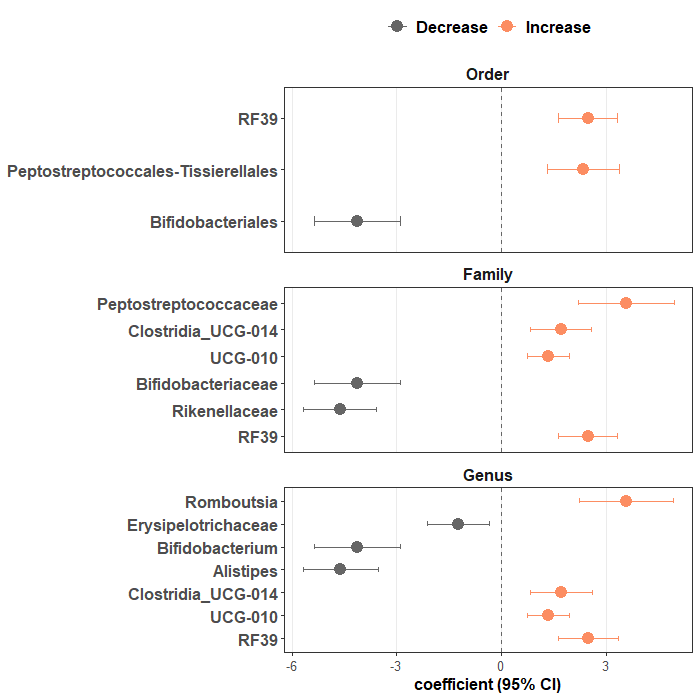
**

**Supplementary Figure 3. Exploratory differential abundance analysis between SM and SM+LF using MaAsLin3.**Forest plots display MaAsLin3 coefficients (β) for taxa from phylum to genus level comparing SM+LF relative to SM. Positive coefficients indicate enrichment in SM+LF, whereas negative coefficients indicate depletion. Points represent estimated coefficients, and horizontal bars denote 95% confidence intervals.

**
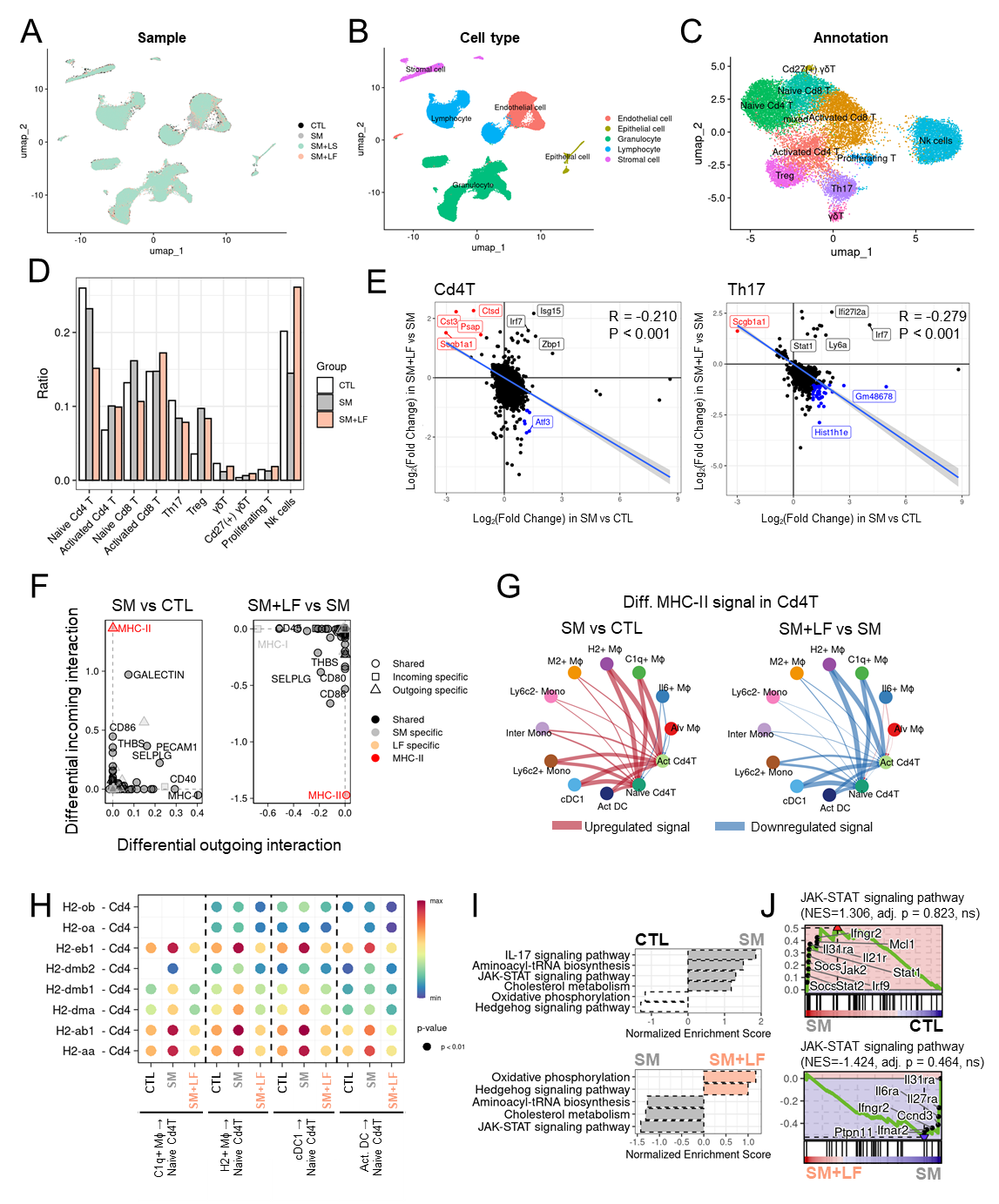
**

**Supplementary Figure 4.** **Single-cell sequencing analysis on the lung tissues from the smoking-induced mouse emphysema model and subsequent analysis of Cd4T**

(a) Uniform Manifold Approximation and Projection (UMAP) representing single cells grouped into samples. (b) UMAP representing single cells grouped into cell types. (c) UMAP of T and NK cells from control, smoking, and smoking with LF groups. Each color represents distinct cell types. (d) The ratio of T and NK subtype cells in each sample. The y-axis of the bar plot indicates the ratio of cell types, while the x-axis of the plot represents the cell types. (e) Comparing differentially expressed genes (DEGs) of Cd4T and Th17 between the control and smoking models, with DEGs between the smoking and smoking with LF models. (f) Relative changes in outgoing and incoming ligand–receptor interactions between Cd4T in each sample. The relative intensity of outgoing ligand–receptor pairs in the smoking to control models (left) and the smoking with LF to smoking models (right) was plotted on the x-axis. (g) Differential strength of intercellular MHC-II towards Cd4T between smoking and control (left) conditions and differential strength between the smoking with LF and smoking models (right) is plotted using a circular plot. The width of arrows is weighted by their maximal intensity. Upregulated interactions are colored in red, and downregulated interactions in blue. (h) Intercellular MHC-II signaling intensity is plotted using a bubble plot. (i) GSEA of Cd4T between the control and smoking models (upper) and between the smoking and smoking with LF models (lower). Pathways with dotted lines do not exhibit significant q values. (j) GSEA plots of the JAK-STAT signaling pathway in Cd4T.


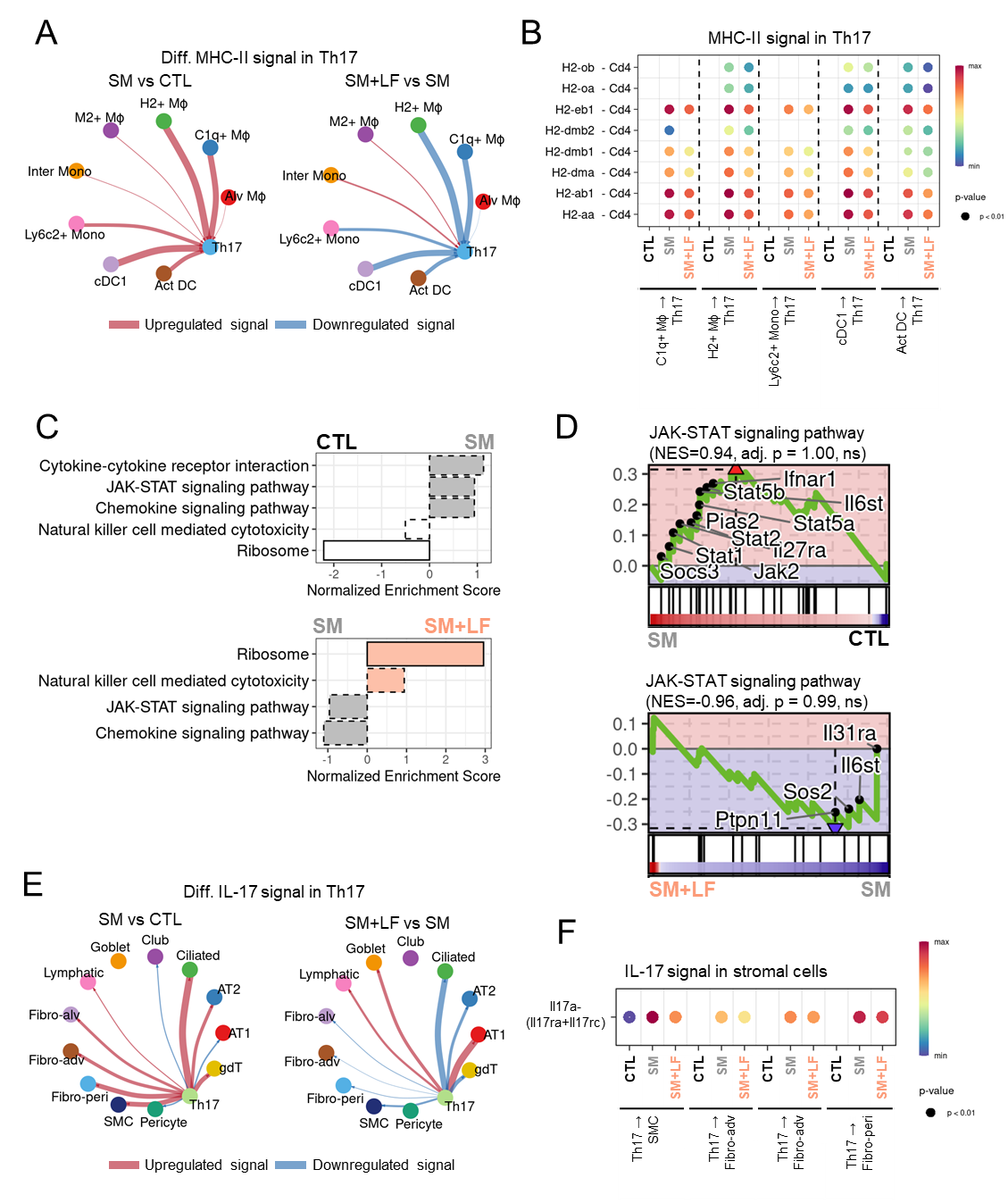


**Supplementary Figure 5.** **GSEA and CCI analysis of Th17**

(a) Differential strength of intercellular MHC-II signaling toward Th17 between the smoking and control (left) conditions, and differential strength between smoking with LF and smoking (right) conditions is plotted using the circular plot. (b) Intercellular MHC-II signaling intensity is plotted using the bubble plot. (c) GSEA of Th17 between the control and smoking models (upper) and between the smoking and smoking with LF models (lower). Pathways with dotted lines do not exhibit significant q values. (d) GSEA plots of the JAK-STAT signaling pathway in Th17 T cells. (e) Differential strength of intercellular IL-17 signaling from Th17 between the smoking and control (left) conditions and differential strength between the smoking with LF and smoking (right) conditions is plotted on a circular plot. (f) Intercellular IL-17 signaling intensity is plotted using a bubble plot.


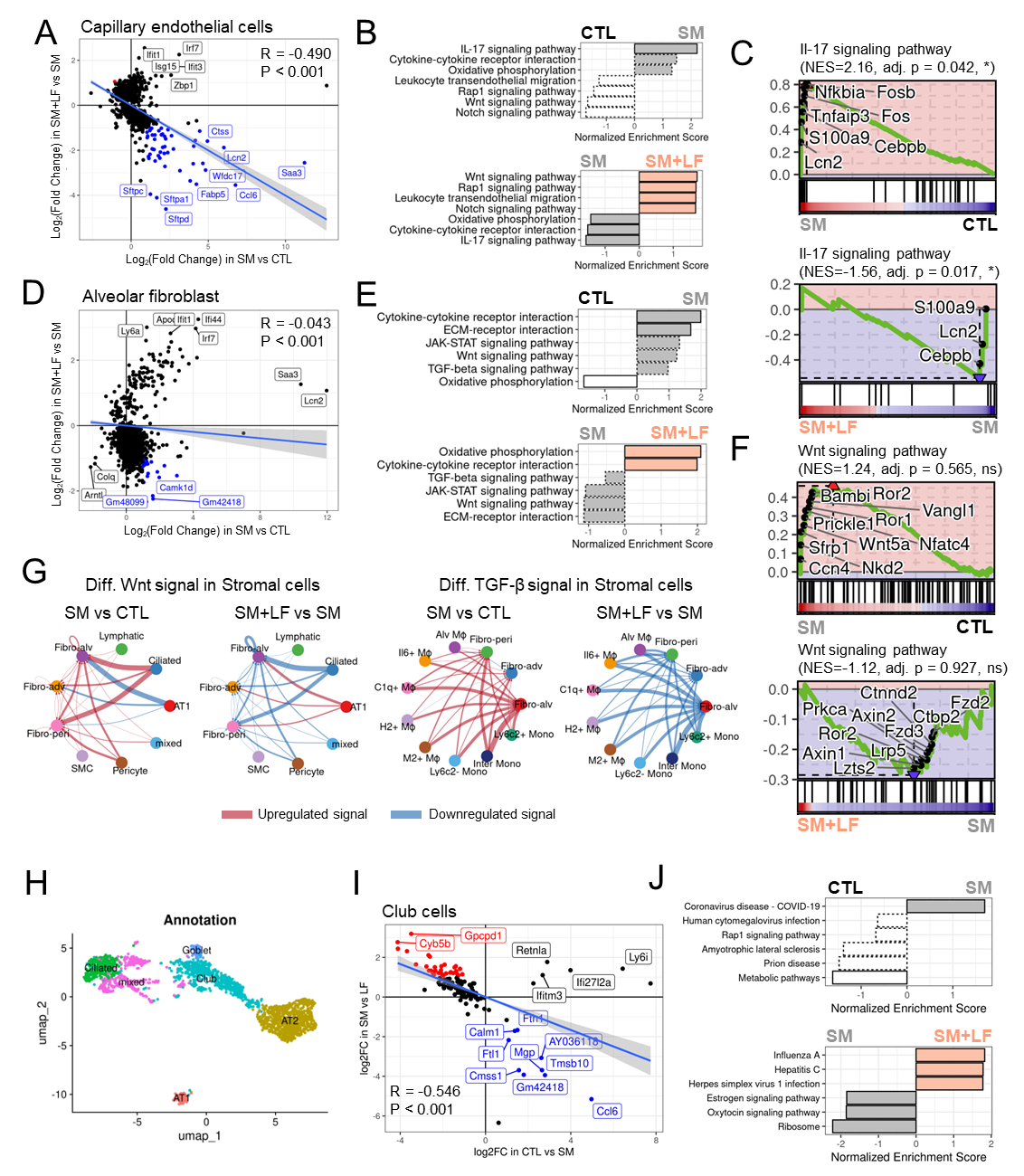


**Supplementary Figure 6.** **DEG, GSEA, and CCI analysis of endothelial and stromal cells**

(a) Comparing differentially expressed genes (DEGs) of capillary endothelial cells between the control and smoking models and between the smoking and smoking with LF models. (b) Gene set enrichment analysis (GSEA) of capillary endothelial cells between the control and smoking models (upper) and between the smoking and smoking with LF models (lower). (c) GSEA plots of the IL-17 signaling pathway in capillary endothelial cells. (d) Comparing the DEGs of the alveolar fibroblasts between the control and smoking models with the alveolar fibroblast of alveolar macrophages between smoking and smoking with the LF model. (e) GSEA of alveolar fibroblasts between the control and smoking models (upper) and between the smoking and smoking with LF models (lower). (f) GSEA plot of the Wnt signaling pathway in the alveolar fibroblasts. (g) Differential intensity of Wnt and TGF-β signaling toward stromal cells plotted using a circular plot. (h) UMAP of epithelial cell (i) Comparing the DEGs of the club cells between the control and smoking models with alveolar fibroblast of alveolar macrophages between smoking and smoking with LF model. (j) GSEA of club cells between the control and smoking models (upper) and between the smoking and smoking with LF models (lower).


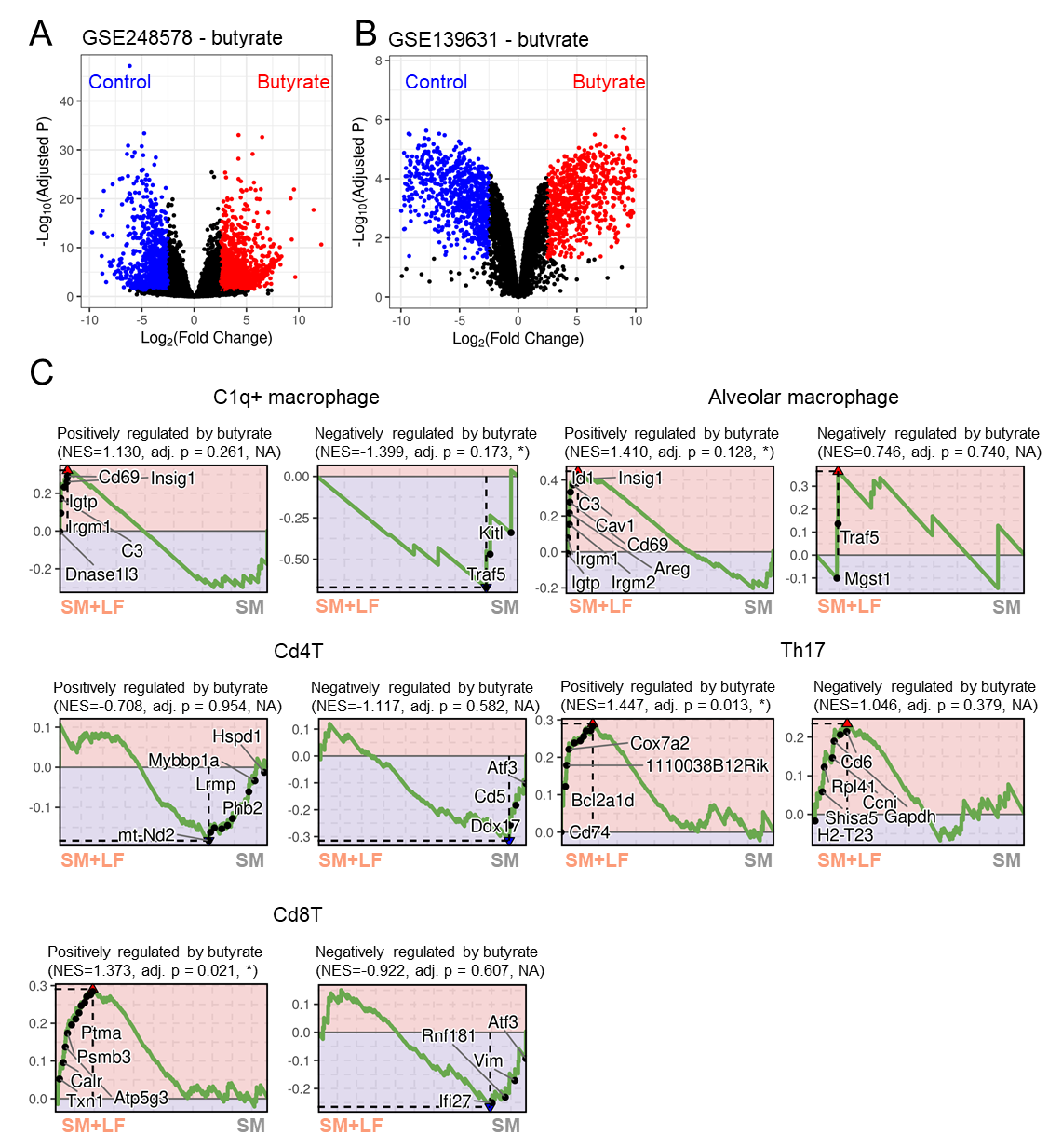


**Supplementary Figure 7. Butyrate-responsive transcriptional programs and their concordance with LF-associated immune remodeling signatures**

(A–B) Volcano plots depicting differential gene expression in publicly available transcriptomic datasets comparing butyrate-treated vs. control conditions (GSE248578, panel A; GSE139631, panel B). Each point represents a gene plotted by log₂(fold change) on the x-axis and -log10(Benjamini–Hochberg–adjusted P value) on the y-axis. Genes upregulated by butyrate are shown in red, downregulated genes in blue, and non-significant genes in black (multiple-testing adjustments as provided in the original datasets). (C) Cross-dataset gene set enrichment analysis (GSEA) linking published butyrate-regulated gene sets to immune cell states identified in the current lung single-cell RNA-seq analysis. For each indicated population (C1q⁺ macrophages, alveolar macrophages, CD4 T cells, Th17 cells, and CD8 T cells), enrichment plots are shown for gene sets positively or negatively regulated by butyrate, with the normalized enrichment score (NES) and adjusted P value reported in each panel. Representative leading-edge genes contributing to the enrichment signal are annotated.


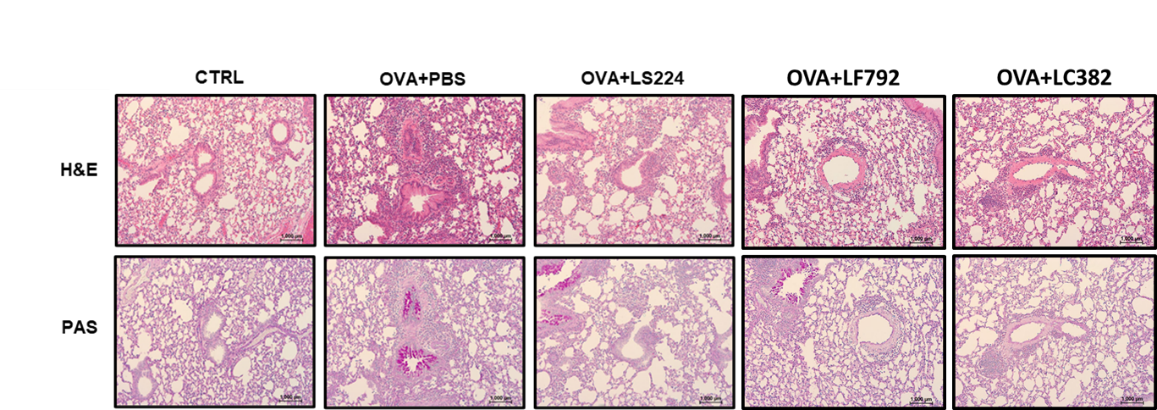


**Supplementary Figure 8. Representative lung histology from an OVA-induced allergic airway inflammation model used for the stepwise prioritization of *Lactobacillus* candidates.**Representative H&E-stained lung sections are shown for the control group, the OVA-challenged group, and the OVA-challenged groups treated with each of the shortlisted *Lactobacillus* isolates. These histological images served as supportive qualitative evidence during the stepwise prioritization process to identify candidates with the potential to attenuate airway inflammation ^3^ . The three strains subsequently advanced to PMAS/COPD-oriented evaluation (LS, LC, and LF) are presented. Scale bar: 1000 µm.

**
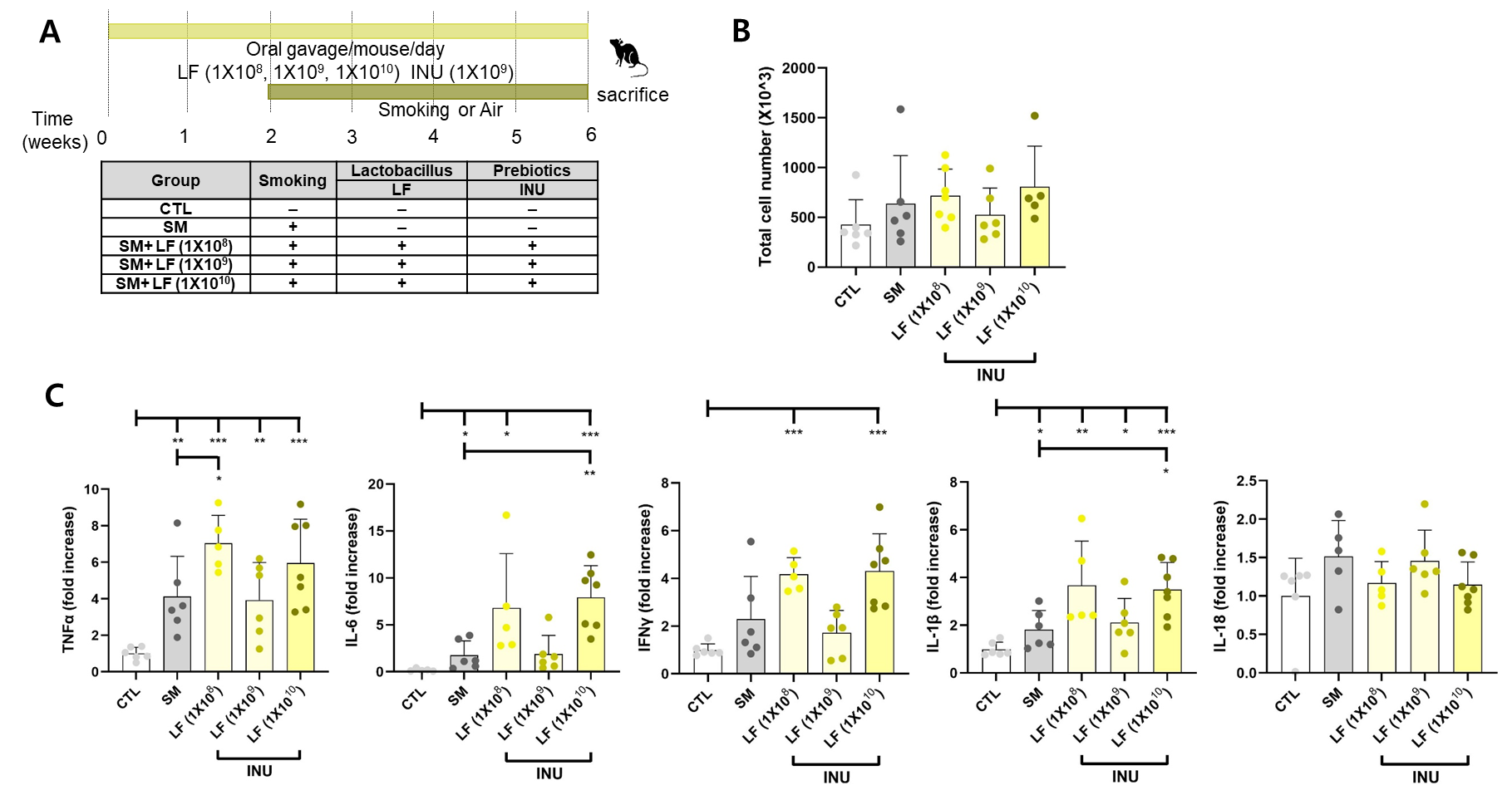
**

**Supplementary Figure 9. Pilot dose-ranging synbiotic experiment supports the selection of LF 1×10⁹ CFU/mouse/day for subsequent in vivo studies.**
Mice were assigned to the following groups: CTL, SM, SM+INU+LF 1×10⁸, SM+ INU +LF 1×10⁹, or SM+inulin+LF 1×10¹⁰. (A) Experimental design and treatment groups. Mice were exposed to cigarette smoke (SM) or room air (CTL) and received LF by oral gavage at 1×10⁸, 1×10⁹, or 1×10¹⁰ CFU/mouse/day with concomitant inulin (INU) administration (oral, 20 mg/200 ul/mouse, 5 times/week) during the exposure period (6 weeks) as indicated. (B) Total bronchoalveolar lavage (BAL) cell counts at the endpoint. (C) Lung mRNA expression of inflammatory cytokines measured by qPCR, including Tnfα, Il6, and Ifng; additional targets, if shown, are indicated in the panel labels. Data are presented as fold change relative to CTL after normalization to 18S. Each dot represents one mouse; bars indicate mean ± SD. Sample sizers were n = 6 for CTL, n = 6 for SM, n = 8 for SM+inulin+LF 1×10⁸, n = 8 for SM+inulin+LF 1×10⁹, and n = 8 for SM+inulin+LF 1×10¹⁰. Statistical comparisons were performed using a two-sample t-test. *P < 0.05, **P < 0.01, ***P < 0.001.

**
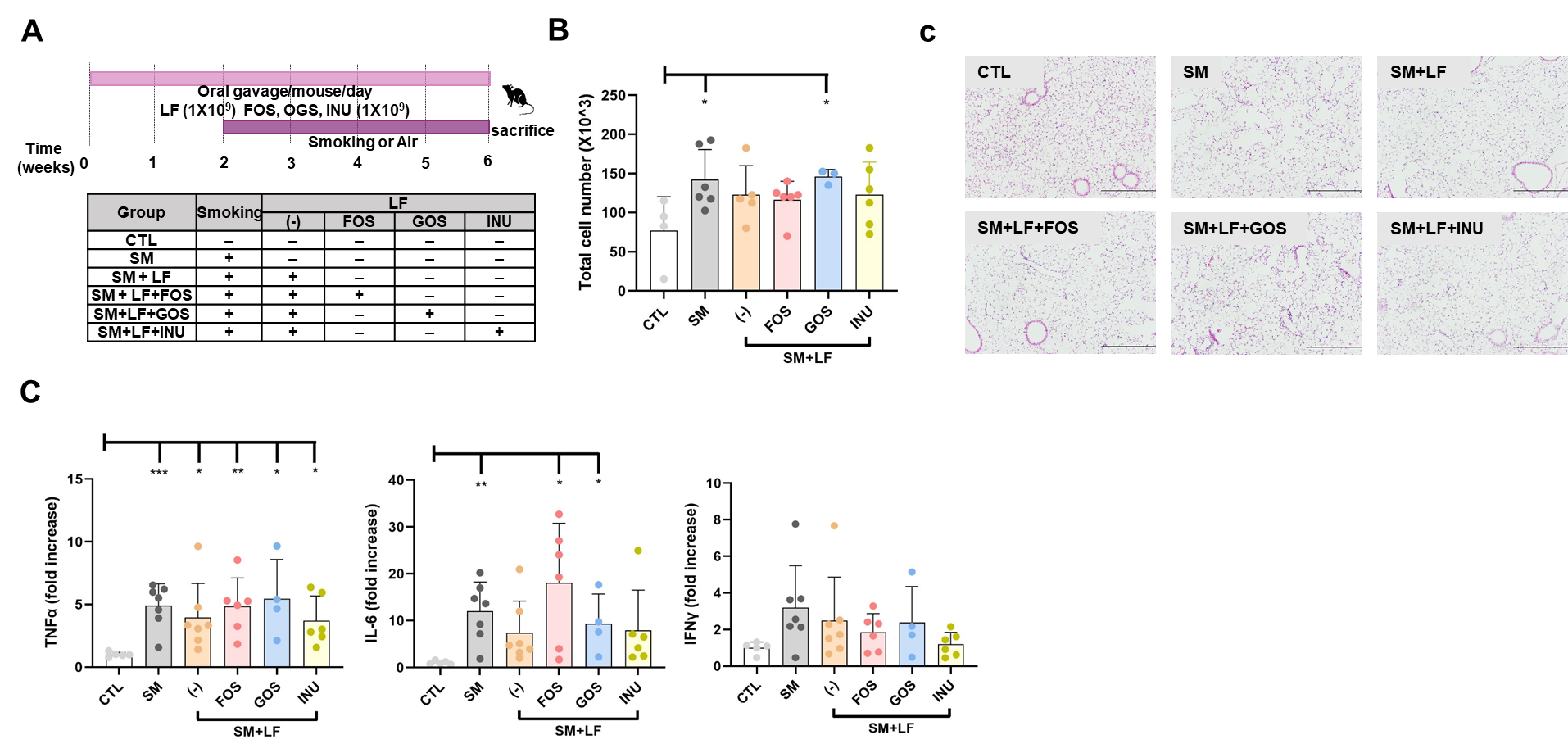
**

**Supplementary Figure 10. Pilot evaluation of prebiotic add-on to LF (1×10⁹ CFU/mouse/day) in a smoke-exposed model.**(A) Experimental design and treatment groups. Smoke-exposed mice received LF by oral gavage (1×10⁹ CFU/mouse/day) either alone or in combination with one of the following prebiotics: fructooligosaccharide (FOS), galactooligosaccharide (GOS), or inulin (INU). These were administered via oral gavage at 20 mg in 200 µL/mouse/day for 6 weeks, as indicated. (B) Total BAL cell counts at the endpoint. (C) Representative H&E-stained lung sections. (D) Lung inflammatory cytokine transcripts measured by qPCR (*Tnfα*, *Il6*, and *Ifng*), expressed as fold change relative to the CTL group after normalization to a housekeeping gene (e.g., 18S). Each dot represents one mouse; bars indicate mean ± SD. Sample sizes were n = 5 for CTL, n = 7 for SM, n = 6 for SM+FOS+LF 1×10^9^, n = 4 for SM+GOS+LF 1×10^9^, and n = 6 for SM+inulin+LF 1×10⁹. *P < 0.05, **P < 0.01, ***P < 0.001.

**References**

1. Liu, M. et al. Emerging Biological Functions of IL-17A: A New Target in Chronic Obstructive Pulmonary Disease? *Front Pharmacol* **12**, 695957 (2021).

2. Mai, J. et al. Interleukin-17A Promotes Aortic Endothelial Cell Activation via Transcriptionally and Post-translationally Activating p38 Mitogen-activated Protein Kinase (MAPK) Pathway. *J Biol Chem* **291**, 4939–4954 (2016).

3. Kim, H. S. et al. Multifunctional effects of Lactobacillus sakei HEM 224 on the gastrointestinal tract and airway inflammation. *Sci Rep* **13**, 17918 (2023).
